# Lighting up materials with X-rays: advanced X-ray excited optical luminescence probe at the BM08-XAFS/XRF beamline, SESAME

**DOI:** 10.1107/S2052252526007815

**Published:** 2026-10-06

**Authors:** Latif U. Khan, Abdel R. Hasoneh, Abdalla Ahmad, Can Dokuyucu, Anas Mohammad, Allayth Aldrabee, Muhammad Iqbal, Farouq Al Omari, Almuatasem Al Natsheh, Abid U. Rehman, Maher Shehab, Mustafa AlZubi, Hussein Al-Mohammad, Zahid U. Khan, Felipe da S. M. Canisares, Messaoud Harfouche, Santiago J. A. Figueroa, Fareeha Hameed, Andrea Lausi, Hermi F. Brito

**Affiliations:** a Synchrotron-light for Experimental Science and Applications in the Middle East (SESAME), PO Box 7, Allan19252, Jordan; b Jordan Atomic Energy Commission, Amman11934, Jordan; c Computer Science Department, Princess Sumaya University for Technology (PSUT), Khalil Saket Street, Amman11941, Jordan; d Institute of Chemistry University of São Paulo (USP) 05508-000 São Paulo-SP Brazil; e Research Centre for Greenhouse Gas Innovation University of São Paulo (USP) 05508-030 São Paulo-SP Brazil; f Brazilian Synchrotron Light Laboratory (LNLS) Brazilian Center for Research in Energy and Materials (CNPEM) 13083-970 Campinas São Paulo Brazil; g Institute of Chemistry, University of Campinas, 13083-862 Campinas, São Paulo, Brazil; h Instituto de Física, Universidade de São Paulo (IFUSP), São Paulo, Brazil; UCL, United Kingdom

**Keywords:** XEOL setup, sample environments, XEOL spectroscopy, X-ray scintillators, SESAME, X-ray excitation, emission spectra, hyperspectral mapping

## Abstract

A highly sensitive X-ray excited optical luminescence (XEOL) probe has been developed and commissioned at the end station of the BM08-XAFS/XRF beamline, SESAME, capable of measuring emission spectra in a broad wavelength range from 200 to 950 nm with optical resolution of ∼1.60 nm, under both X-ray beam and laser (UV–Vis/NIR) excitation sources. This experimental setup is equipped with a high-sensitivity spectrometer coupled to a customized sample environment via an optical fiber of 0.22 numerical aperture. The in-house-developed sample environment contains multiple entrance windows for the simultaneous X-ray beam and laser irradiations, and an exit window to a Basler camera coupled via a focusing lens. The real-time monitoring of the emission’s spatial distribution in material under element-specific X-ray excitation and laser irradiation through an integrated XEOL camera, and acquisition of the emission spectra and two-dimensional hyperspectral mapping using an integrated spectrometer, provides a powerful probe for the development of homogeneous ionizing-radiation scintillators. Such materials are critically important for advanced imaging technologies, including X-ray computed tomography. XEOL studies and hyperspectral mapping were performed on selected representative systems/X-ray scintillators including Gd_3_Ga_5_O_12_:Eu^3+^ (GGG:Eu), Gd_2_O_2_S:Tb^3+^, Gd_2_O_3_, semiconductor quantum dots, rare-earth ion (RE^3+^) complexes and organic polymer/RE^3+^ hybrid films to demonstrate the performance of the setup across a broad spectral range from the UV–Vis to NIR regions. The combination of XEOL with X-ray absorption spectroscopy at BM08 enables users to probe optical emission pathways in material and correlate them directly with the electronic structure and chemical environment of the photoemitting centers in the material.

## Abstract 2

An advanced X-ray excited optical luminescence experimental probe has been developed and commissioned at the BM08-XAFS/XRF beamline, SESAME. The setup is now available to users for measuring emission spectra and performing hyperspectral mapping of a wide range of optical materials over the 200–950 nm spectral range, under monochromatic X-ray beam and laser (UV–Vis/NIR) excitation.

## 1. Introduction

X-ray excited optical luminescence (XEOL) is a remarkable X-ray-photon-in and optical-photon-out technique (Sham, 2014 ▸; Lei *et al.*, 2022 ▸; Rogalev & Goulon, 2002 ▸) for exploring optical emission in a wide range of luminescent/X-ray scintillating materials (Sham, 2018 ▸; Wang *et al.*, 2014 ▸). XEOL has been known since the discovery of X-rays, and is primarily employed to detect rare-earth trace metals and visualize X-ray beams. Bianconi *et al.* (1978 ▸) suggested XEOL as an alternative optical detection method for X-ray absorption fine structure (XAFS) measurements (Penner-Hahn, 2026 ▸). Recently, the development of advanced synchrotron light sources with high photon flux, highly focused X-ray beams and ultrafast highly sensitive photon detectors, such as charge-coupled devices (CCDs) (Phillips *et al.*, 2002 ▸), has significantly expanded the applicability of XEOL. These advances have enabled XEOL to be an efficient element site-specific and energy-level-selective technique (Fonseca *et al.*, 2025 ▸) in optical spectroscopy and two-dimensional hyperspectral mapping for probing the optoelectronic structure (Khan *et al.*, 2026 ▸) of advanced optical materials.

X-ray absorption spectroscopy (XAS) and XEOL have been offered a powerful combination probe (Wang *et al.*, 2014 ▸; Hageraats *et al.*, 2021 ▸; denBoer, 2003 ▸) to unlock the lattice defects, local chemical environment and optoelectronic structure of a photoemitter’s sites in materials (Fonseca *et al.*, 2025 ▸). Time-resolved XEOL (TR-XEOL) (Huang *et al.*, 2024 ▸; Regier *et al.*, 2025 ▸) has provided additional selectivity for XAFS to unlock the detailed optoelectronic structure of the photoemitter sites (Vogt & Smolentsev, 2022 ▸). Therefore, XEOL can be found at most XAS beamlines of advanced synchrotron light sources. XEOL has been currently added as a two-dimensional imaging technique for microfocus and nanofocus X-ray microscopy and spectroscopy beamlines (Teixeira *et al.*, 2023 ▸). Examples include XEOL at the VLS-PGM beamline of the Canadian Light Source (Hu *et al.*, 2007 ▸), a steady-state XEOL spectroscopy setup at the XAS beamline P65 of PETRA III (Levcenko *et al.*, 2022 ▸), an XEOL microprobe for spectroscopy and 2D imaging at the TARUMA station of the CARNAÚBA X-ray nanospectroscopy beamline of Sirius-LNLS, Brazil (Teixeira *et al.*, 2023 ▸), and an XEOL/TR-XEOL facility on the I18 microfocus beamline at the Diamond Light Source, the UK national synchrotron facility (Taylor *et al.*, 2013 ▸). The 23A X-ray nanoprobe beamline of the Taiwan Photon Source (TPS) has remarkably integrated XEOL with TR-XEOL (Huang *et al.*, 2024 ▸). They used an iHR550 Horiba spectrometer equipped with a CCD (Syncerity BI UV–Vis) and photomultiplier tube (PMT) for the XEOL measurement and an iHR320 Horiba spectrometer equipped with a Hamamatsu C10910 streak camera for the TR-XEOL measurement, using an inter-switching optical fiber (Huang *et al.*, 2024 ▸). The XEOL setup at the HERMES beamline of SOLEIL has remarkably combined XEOL and XAS for correlative imaging on the nanoscale level (Hageraats *et al.*, 2021 ▸). In XEOL nano­imaging, the synchrotron radiation tunable nanofocused X-ray beam induces luminescence in a highly localized region of the sample. By raster-scanning the sample relative to the X-ray beam and recording both XEOL and XAS signals at each position, hyperspectral XEOL and XAS datasets can be acquired simultaneously with high spatial resolution (Lin *et al.*, 2019 ▸). These combined spectroscopic measurements provide detailed insight into the optoelectronic structure and chemical coordination environment of the photoemitting metal sites in the material.

XEOL is a site-selective and core-level transition-selective process (Song *et al.*, 2024 ▸; Penner-Hahn, 2026 ▸), where the energy of the synchrotron radiation X-ray can be tuned to the selective core-level transition of the photoemitter (Rezende *et al.*, 2016 ▸), giving site-specific optical emission to gain insight on the local site of the photoemitting metal ion. For instance, XEOL emission probes the *d*–*d* transitions of the photoemitter (*e.g.* Mn^2+^ doped in ZnSe) (Gul, Khan, Galani *et al.*, 2025 ▸; Gul, Khan, Khan *et al.*, 2025 ▸) and interconfigurational 4*f*–5*d* (Li *et al.*, 2024 ▸) or 4*f*-intraconfigurational transitions of the photoemitting divalent/trivalent rare-earth (RE^2+/3+^) ions, such as Eu^2+^/Eu^3+^ incorporated in a BaAl_2_O_4_ host (Khan *et al.*, 2025 ▸). In quantum confined semiconductors, the XEOL emission originates from the defects and/or surface states (Saurabh *et al.*, 2025 ▸) in the material (Wang *et al.*, 2025 ▸). The intensity of the specific XEOL emission bands from the photoemitting sites, such as Eu^2+/3+^, can be measured as a function of the excitation energy (Khan *et al.*, 2025 ▸), which is increased when increasing the excitation energy of the monochromatic X-ray beam, providing an ideal platform to measure XAFS (Bianconi *et al.*, 1978 ▸) in XEOL mode.

XEOL is a phenomenon involving multiple energy-transfer processes (Song *et al.*, 2024 ▸; Novais *et al.*, 2012 ▸), originated via the absorption of X-ray photons by absorbing atoms within the material, resulting in the generation of core holes and energetic electrons (photoelectrons and Auger electrons) (Rezende *et al.*, 2016 ▸; Sham, 2022 ▸). These energetic electrons subsequently induce further ionization and excitation events throughout the material. Through thermalization and inelastic scattering processes, the absorbed energy is transferred to the excited states of luminescent centers (*e.g.* RE^3+^ ions), followed by radiative relaxation that produces XEOL emission (Khan *et al.*, 2025 ▸). XEOL has been proposed as an effective technique for obtaining site- and local-chemical-environment-sensitive information on multisite systems because of its sensitivity to surface defects, local chemical environments and the local symmetry surrounding the luminescent centers, such as Eu dopant sites in BaAl_2_O_4_ (Chayanun *et al.*, 2025 ▸; Huang *et al.*, 2024 ▸; Penner-Hahn, 2026 ▸; Khan *et al.*, 2025 ▸). The site selectivity and depth sensitivity of XEOL arise from the combined effects of X-ray absorption depth and the thermalization of secondary electrons generated during core-hole relaxation. The spatial extent of these secondary-electron cascades, which is governed by the electron inelastic mean free path, determines the distribution of electron–hole excitations responsible for luminescence generation (Sham, 2022 ▸; Rogalev & Goulon, 2002 ▸). This intrinsic sensitivity makes XEOL particularly advantageous for investigating complex, heterogeneous, environmental and multicomponent materials that are often difficult to probe using conventional structural characterization techniques.

In this work, we unlocked the capabilities of a new high-sensitivity XEOL setup developed at the BM08-XAFS/XRF beamline (Harfouche *et al.*, 2022 ▸), SESAME, and performed considerable spectral-resolution XEOL and micrometre-spatial-resolution spectral-mapping measurements in a broad spectral range. The main advantage of this setup is the in-house-developed customized 3D-printed sample environment, which allows simultaneous measurements of XEOL and XAS without further sample alignment and subsequent photoluminescence (PL) under laser and UV source irradiation. We performed X-ray induced luminescence and spectral-mapping studies on selective optical materials, such as X-ray scintillators, quantum dots (QDs), Eu^3+^ complexes/thin films, Gd_2_O_3_, BaAl_2_O_4_:Eu^2/3+^ and CeCl_3_·6H_2_O, to demonstrate the setup’s capability in the UV–Vis to NIR wavelength ranges.

## 2. Experimental section

### 2.1. XEOL experimental setup

The XEOL experimental setup at the end station of the BM08-XAFS/XRF beamline (Harfouche *et al.*, 2022 ▸), SESAME, contains an in-house-developed customized 3D-printed sample environment, an optical fiber and an XEOL spectrometer. The customized sample environment contains multiple entrance windows for the X-ray beam and laser, to measure XAFS and XEOL simultaneously without further sample alignment and subsequent PL spectral measurement. The sample environment is equipped with a Basler ace GigE camera and LMVZ540 focusing lens, Kowa, of 40 mm focal length. This is integrated into the XEOL setup to capture real-time digital and fluorescent images of the optical materials under white light and irradiation with an X-ray beam/laser, using *Pylon* software. The optical emission from the sample is collected through a stainless steel BX-covered solarization resistant optical fiber (Ocean Insight) of 600 mm core diameter and 0.22 numerical aperture, covering the UV–Vis and NIR range (200–1100 nm). A collimating lens (74-UV) of 200–2000 nm wavelength range is connected to the optical fiber, which controls the field of view (FOV) of the emission collecting from a sample. In addition, UV–Vis filters of desired cutoff wavelength can be loaded inside the in-house-developed housing that is connected to the end of the optical fiber looking at the sample. The selective wavelength cutoff filter can be used to avoid the stray scattering of UV–Vis light from the laser source. The optical fiber is connected to a QE Pro-ABS high-sensitivity spectrometer (Ocean Insight, preconfigured model) through SMA 905 connections. The spectrometer operates in the wavelength range from 200 to 950 nm with integration times between milliseconds and seconds and an optical resolution of ∼1.60 nm (full width at half-maximum, FWHM). The spectrometer is equipped with a Hamamatsu scientific grade back-thinned thermal electrical cooled 1024 × 58 elements CCD array detector.

### 2.2. XEOL measurements

**Figure 1:**
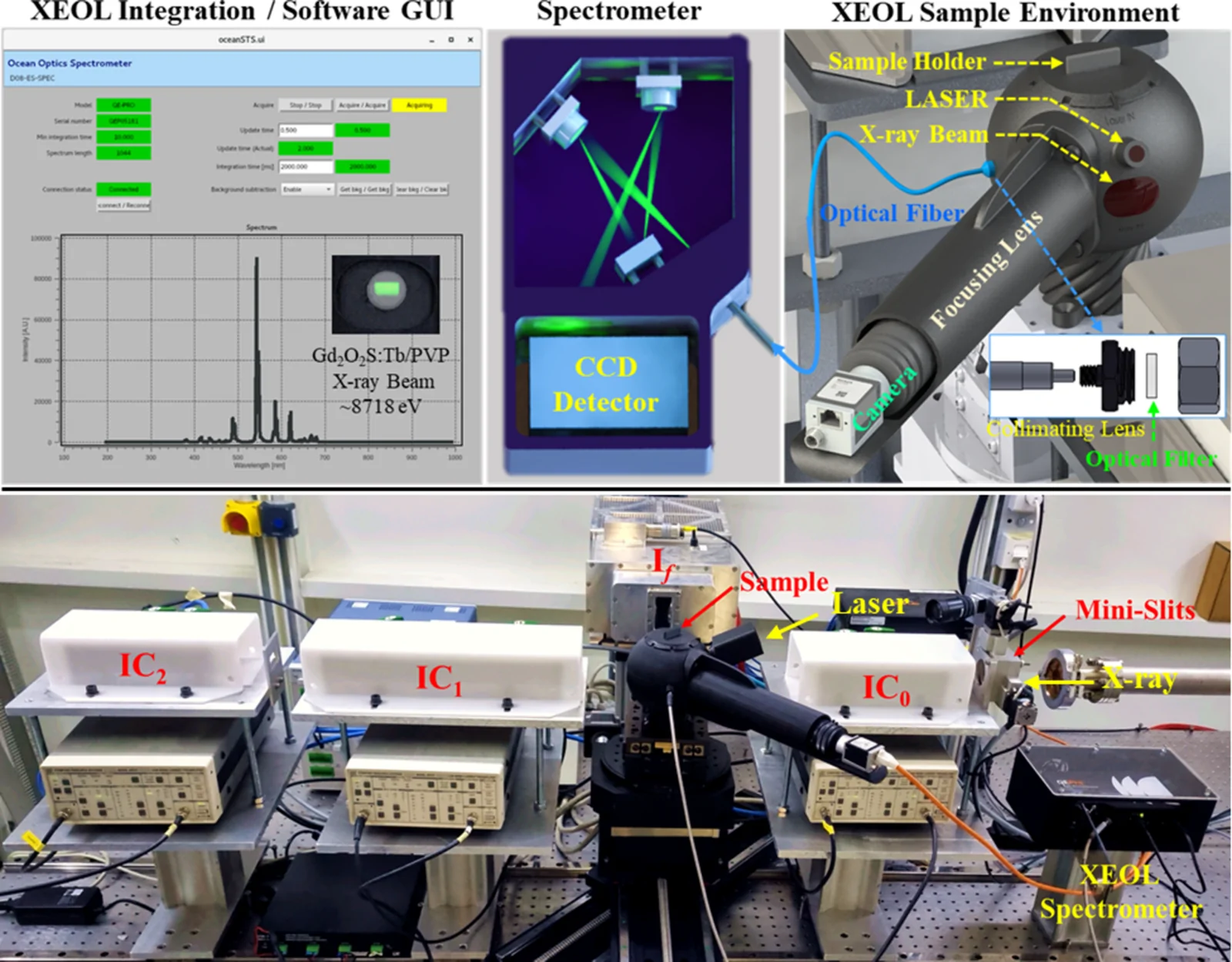
General view of the XEOL experimental setup (top) and end station of the BM08-XAFS/XRF beamline (bottom), SESAME. A sample environment with an optical fiber and collimating lens for collecting the luminescence signals (top right) and a spectrometer for collecting the luminescence spectra under irradiation with X-ray/laser/UV sources (top middle) are shown. Digital luminescence images are captured using a Basler camera.

XEOL spectra of the optical materials are acquired under irradiation with a monochromatic X-ray beam of specific energies above and below the *L* and *K* edges of the specific metals, according to the energy range (4700–30000 eV) of the BM08-XAFS/XRF beamline (Harfouche *et al.*, 2022 ▸), SESAME, operating at 2.5 GeV in ‘decay’ mode and a maximum electron current of 300 mA. The BM08 beamline provides a photon flux of ∼5 × 10^11^ photons s^−1^ with a minimum beam size of 2 × 2 mm (horizontal × vertical). The sample is aligned at a 45° angle to the incoming monochromatic X-ray beam and is loaded into the sample holder of a customized sample environment, which allows easy changing of the samples from the top of the environment (Fig. 1 ▸). The optical emission from the sample is collected through an optical fiber, positioned at 90° to the X-ray beam axis and closely facing the sample, which is connected at the other end to the XEOL spectrometer. The XEOL spectra and hyperspectral-mapping data can be acquired in the spectral range from 200 to 950 nm, with integration times between milliseconds and seconds and an optical resolution of ∼1.60 nm (FWHM). Multiple accumulated spectra can be measured and averaged for each sample using commercial *OceanView* software and a graphical user interface (GUI) developed in-house in *PyQt/Qt Designer* and the high-level programming language Python. The data format for saving spectral data is a TXT file with binary wavelength and intensity columns, and spectral-mapping data are saved in HDF5 format.

## 3. Results and discussion

The XEOL probe is designed for the comprehensive luminescence study of a wide range of optical materials, ranging from luminescent rare-earth phosphors to semiconductor QDs, perovskites nanocrystals and fluorescent metallic nanoparticles. The sample environment is carefully engineered to facilitate simultaneous monochromatic X-ray beam and optical excitation to simultaneously measure XEOL/PL and XAFS (Fig. 1 ▸). The luminescence emitted from the sample is efficiently collected through an optical fiber coupled to the XEOL spectrometer. The solarization resistance, stainless steel BX jacket and high numerical aperture of the optical fiber ensure maximum optical photon collection and minimize spectral distortion during XEOL measurement. In addition, the collimating lens connected to the optical fiber controls the FOV of the emission collecting from a sample at proper distance. The real-time images of the optical materials under X-ray irradiation via an integrated camera allow visual monitoring of the luminescence properties, spatial distribution of the emitted light and potential inhomogeneities in the sample (Fig. 1 ▸). Moreover, the XEOL probe is optimized for spectral mapping of optical materials with micrometre-scale spatial resolution under focused laser and X-ray irradiation.

The XEOL probe has been available to users since July 2023, and has been actively receiving proposals for high-resolution XEOL and PL spectral measurements under simultaneous irradiation with an X-ray beam and a laser source. The XEOL setup has been successfully integrated into the control console of the BM08-XAFS/XRF beamline, enabling synchronized operation with the beamline instrumentation. This integration allows smooth control and data acquisition within the existing beamline framework (Fig. 1 ▸). We are continuously improving this capability, with future upgrades planned to enable full XEOL and µ-XRF 2D mapping at the BM08-XAFS/XRF beamline upon implementation of a microfocus X-ray beam. Notably, we have already demonstrated 2D mapping capabilities using the existing X-ray beam in combination with a focused laser (∼200 µm spot size). Using the XEOL setup, we successfully performed laser-excited fluorescence 2D mapping in step-by-step scanning mode, demonstrating the robustness and flexibility of the system for spatially resolved optical spectroscopy.

To further ensure the synchronized data acquisition, we have also developed an in-house trigger generator. This device produces trigger signals upon command from the Experimental Physics and Industrial Control System (EPICS), enabling precise synchronization with the spectrometer. The system allows spectral acquisition with integration times ranging from milliseconds to seconds (see the supporting information and Fig. S1). In addition, we have developed a dedicated GUI using *PyQt/Qt Designer* and Python. This custom software provides flexible instrument control and data acquisition, and is specifically designed to facilitate automated XEOL 2D spectral mapping and future implementation of XAFS measurement in XEOL mode. To demonstrate the performance and versatility of the XEOL setup, we conducted representative studies on QDs, rare-earth phosphor-based X-ray scintillators, trivalent rare-earth (RE^3+^) complexes and thin films, highlighting the system’s capability for high-resolution, synchronized and spatially resolved optical spectroscopy and imaging/mapping under X-ray and laser excitation.

### 3.1. XEOL study of X-ray scintillators

Scintillators are luminescent materials that convert high-energy ionizing radiation, such as X-rays, into optical photons from the UV–Vis to NIR spectral region. X-ray imaging techniques such as X-ray microtomography employ scintillators coupled with microscope objective lenses and CCD cameras, which has become a successful approach for achieving micrometre-scale spatial resolution. Thin-film X-ray scintillators (Mian *et al.*, 2022 ▸), such as Eu^3+^-doped gadolinium gallium garnet (GGG:Eu), play a crucial role in high-resolution X-ray imaging and and provide higher spatial resolution compared with conventional powder screens. The red-emitting GGG thin-film scintillator used in this work was purchased from the European Synchrotron Radiation Facility (ESRF) and is currently employed in the detection system of the Beamline for Tomography (ID10-BEATS), SESAME. Detailed characterization of the GGG scintillator has previously been reported by the ESRF research group (Martin & Koch, 2006 ▸).

**Figure 2:**
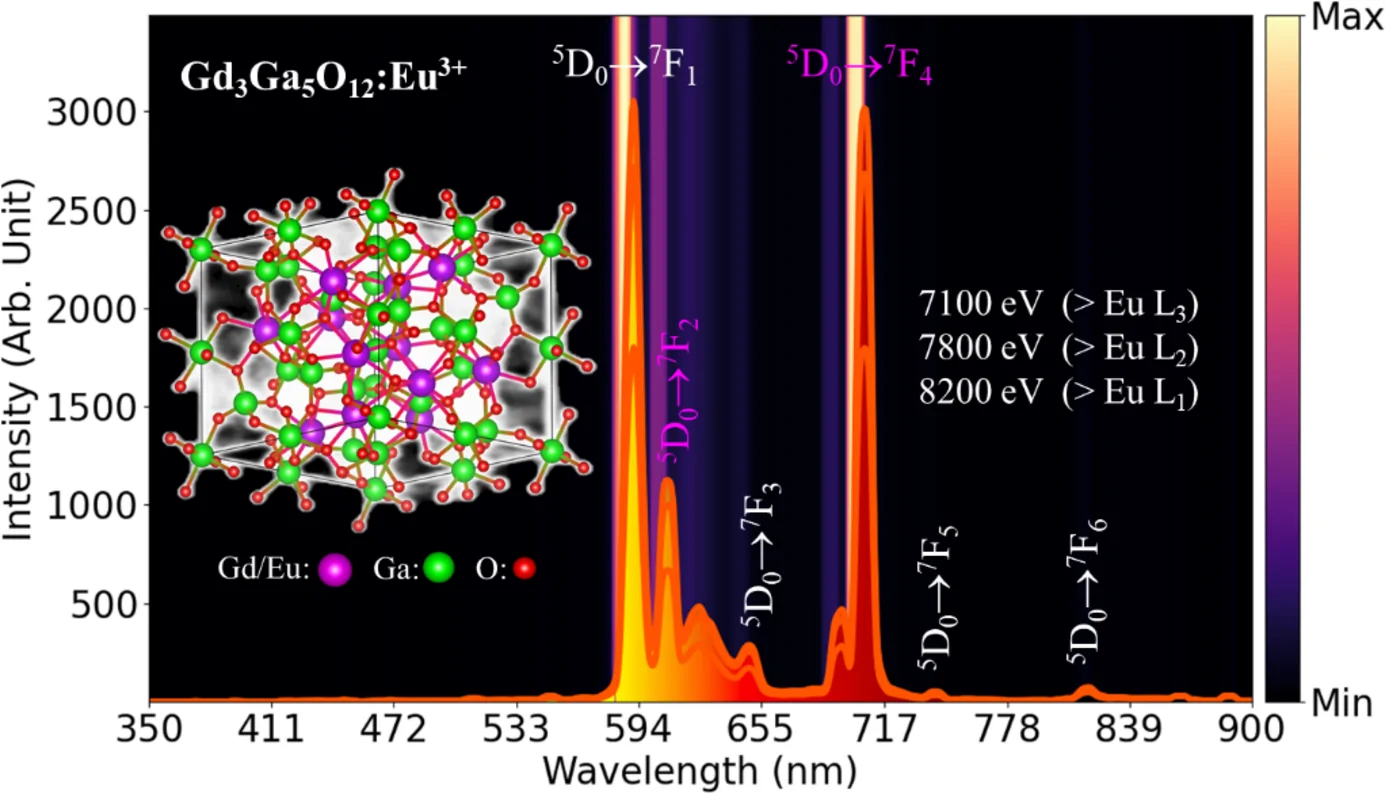
XEOL spectra of the Gd_3_Ga_5_O_12_:Eu^3+^ (GGG:Eu) X-ray scintillator (3D structure in inset figure), measured under excitation at 7100, 7800 and 8200 eV monochromatic X-ray energies, above the Eu *L*_3,2,1_ edges (6977, 7617 and 8052 eV, respectively).

XEOL spectra of the GGG:Eu X-ray scintillator (Fig. 2 ▸) were measured at room temperature (∼300 K) under excitation with a monochromatic X-ray beam of selective energies 7100, 7800 and 8200 eV above the Eu *L*_3,2,1_ edges. Each spectrum was collected by averaging five accumulated scans using the XEOL spectrometer with an integration time of 5 s. The XEOL spectra of the GGG:Eu scintillator displayed narrow emission lines corresponding to the ^5^D_0_ → ^7^F_*J*_ transitions (where *J* = 1–6) of the Eu^3+^ ion. The successful measurement of the weak ^5^D_0_ → ^7^F_6_ forced electric dipole (FED) transition (Fig. 2 ▸) in the NIR region showed the capability and sensitivity of the XEOL spectrometer at the BM08-XAFS/XRF beamline, with extended detection range in the NIR region until 950 nm. This transition is not often measured by conventional spectro­fluoro­meters, which are equipped with blue-sensitive and classic red-sensitive Hamamatsu R928 PMTs with very low sensitivity in this spectral region.

The ^5^D_0_ → ^7^F_1_ transition is an allowed magnetic dipole transition, which is insensitive to the ligand-field effect. The intensity of this transition is largely independent of the chemical environment, mainly depending on the refractive index of the material, and displays almost constant intensity for all the Eu^3+^ compounds. The ^5^D_0_ → ^7^F_2_ transition is a FED and dynamic coupling (DC) hypersensitive transition; however, its emission intensity strongly depends on the chemical environment. The ^5^D_0_ → ^7^F_4_ transition is also a FED transition, and most sensitive to lanthanide-ligating-atom bond distances. Accordingly, the very weak intensity of the hypersensitive ^5^D_0_ → ^7^F_2_ FED transition (Fig. 2 ▸) when compared with the magnetic dipole ^5^D_0_ → ^7^F_1_ transition suggested a centrosymmetric chemical environment for the Eu^3+^ sites in the Gd_3_Ga_5_O_12_ host.

In addition, the intensity of the ^5^D_0_ → ^7^F_4_ transition was observed to be abnormally higher for the GGG:Eu phosphor. This abnormality was described earlier by Malta and co-workers (Sá Ferreira *et al.*, 2006 ▸), who put forward the theory that the Eu^3+^ site in a coordination polyhedron with a local point symmetry being slightly distorted from a high one (for instance, D_4*d*_) can cause abnormally high intensity of the ^5^D_0_ → ^7^F_4_ transition. GGG:Eu contains three nonequivalent symmetry sites: D_2_, D_2*h*_ and C_2*ν*_ (Sharma *et al.*, 2015 ▸). However, the similar ionic radii of the Eu^3+^ and Gd^3+^ ions, 1.07 Å and 1.05 Å, respectively, suggest that the Eu^3+^ ion predominantly enters the eightfold dodecahedral Gd^3+^ sites of the Gd_3_Ga_5_O_12_ host and may possess a D_2_ dominant symmetry site. Nevertheless, the Eu^3+^ emission spectral features indicate that the chemical environment of Eu^3+^ in GGG is more symmetric with slight distortion in the local point symmetry sites.

**Figure 3:**
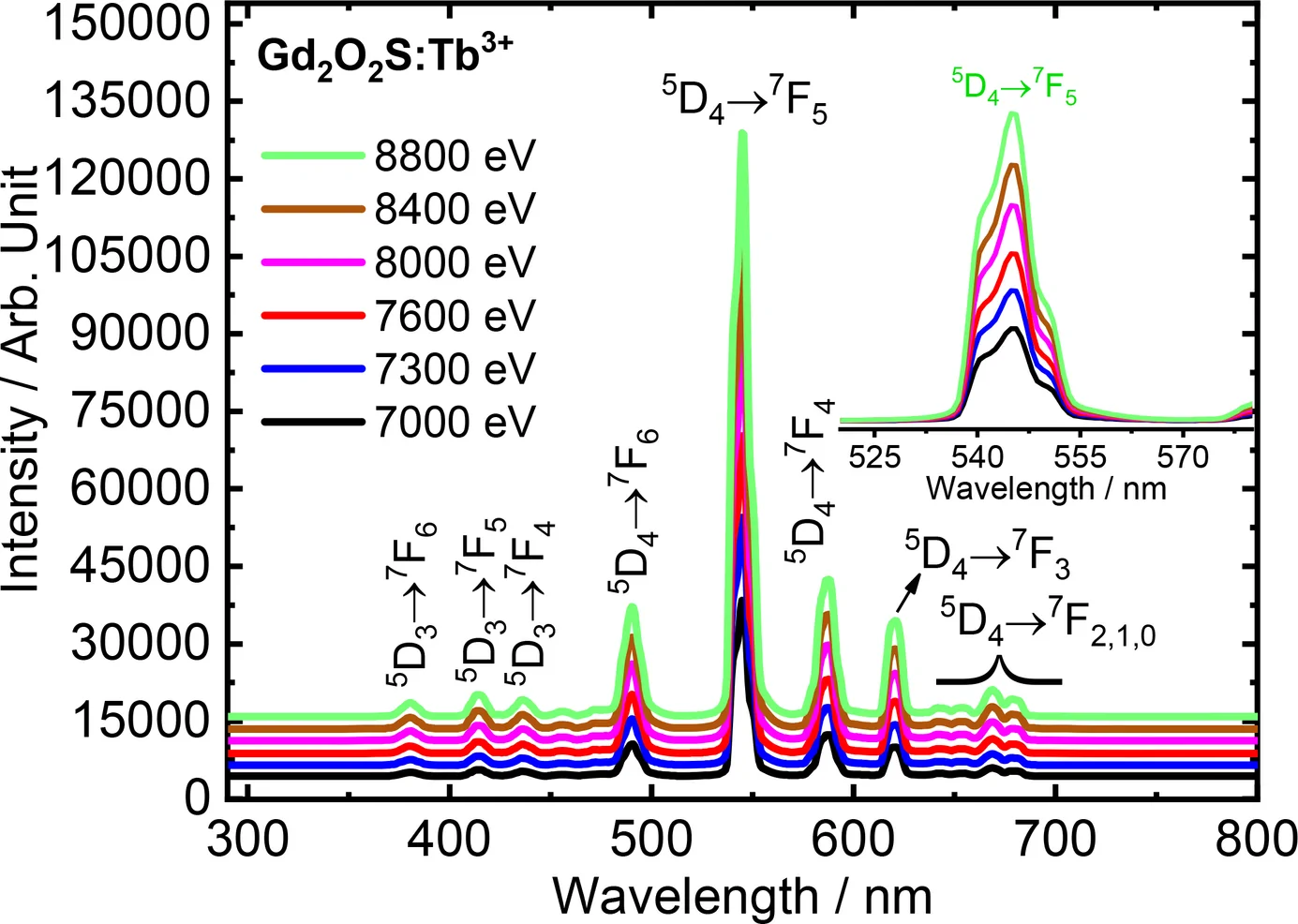
XEOL spectra of the Gd_2_O_2_S:Tb^3+^X-ray scintillator, measured under excitation at 7000–8800 eV monochromatic X-ray energies, around the host Gd *L*_3,2,1_ edges (7243, 7930 and 8376 eV) and photoemitting Tb *L*_3,2,1_ edges (7514, 8252 and 8708 eV). The inset figure shows a magnified spectral region around the emission line from the ^5^D_4_ → ^7^F_4_ transition.

XEOL spectra of the Gd_2_O_2_S:Tb powder scintillator (Fig. 3 ▸) were measured at room temperature under excitation with monochromatic X-ray energies around the host Gd *L*_1,2,3_ edges (8376, 7930 and 7243 eV) and the photoemitter dopant Tb *L*_1,2,3_ edges (8708, 8252 and 7514 eV), measuring ten accumulated scans with an integration time of 5 s for each spectrum. The XEOL spectra show the distinct narrow emission lines assigned to the ^5^D_4_ → ^7^F_*J*_ (*J* = 6–0) transitions of the Tb^3+^ ion (Rodrigues *et al.*, 2020 ▸). The green emission of the Gd_2_O_2_S:Tb^3+^ scintillator under irradiation with the X-ray beam predominantly originates from the dominant high-intensity ^5^D_4_ → ^7^F_5_ transition (∼545 nm). Moreover, narrow emission lines at lower wavelengths were also observed, which are ascribed to the ^5^D_3_ → ^7^F_*J*_ (*J* = 6–4) transitions of the Tb^3+^ ion (Fig. 3 ▸). The detection of well resolved emission lines in the lower-wavelength region manifested the remarkable effectiveness of the XEOL spectrometer in the UV region. However, the absence of Gd^3+^ intraconfigurational 4*f* emission lines in the spectra, even under direct excitation of the Gd 2*s* and 2*p* core electrons, indicates that the absorbed energy is efficiently transferred from Gd^3+^ to the emitting Tb^3+^ ions.

To further probe the effect of excitation of other energy levels on the sensitization of Tb^3+^, the emission spectrum was recorded under X-ray excitation at 7000 eV, *i.e.* below the 2*s* and 2*p* core-level energies of both Gd and Tb (Fig. 3 ▸). The emission spectrum still exhibits considerable emission intensity, suggesting that Tb^3+^ sensitization can also occur via excitation of other electronic levels in the Gd^3+^ host and Tb^3+^ dopant, such as the 4*s* and 4*p* levels (a *p*–*d* electric dipole allowed transition). Although XEOL is a complex process involving the generation of various energetic charge carriers within the material, such as photoelectrons and Auger electrons, accompanied by the formation of core-hole density in the core levels of Gd/Tb due to the high X-ray excitation energy, the subsequent recombination of these electrons and core holes leads to the sensitization of 4*f* excited states of the emitting Tb^3+^ ion. This occurs via multiple energy-transfer pathways [see Fig. S2(*a*)], ultimately resulting in radiative decay from the ^5^D_3,4_ excited states to the ground multiplet ^7^F_*J*_ (*J* = 6–0), giving rise to the characteristic narrow emission lines of the Tb^3+^ ion. In order to gain deeper insight into the sensitization of emitting rare-earth ions in trivalent rare-earth compounds, XEOL studies of typical Eu^3+^ complexes and thin films were performed. These studies also demonstrated that the sensitization of the intraconfigurational 4*f* energy levels of Eu^3+^ can occur through multiple energy-transfer processes, involving the excitation of core *s* and *p* electrons under high-energy X-ray irradiation [see Fig. S2(*b*)].

### 3.2. XEOL studies of Eu^3+^ compounds and thin films

**Figure 4:**
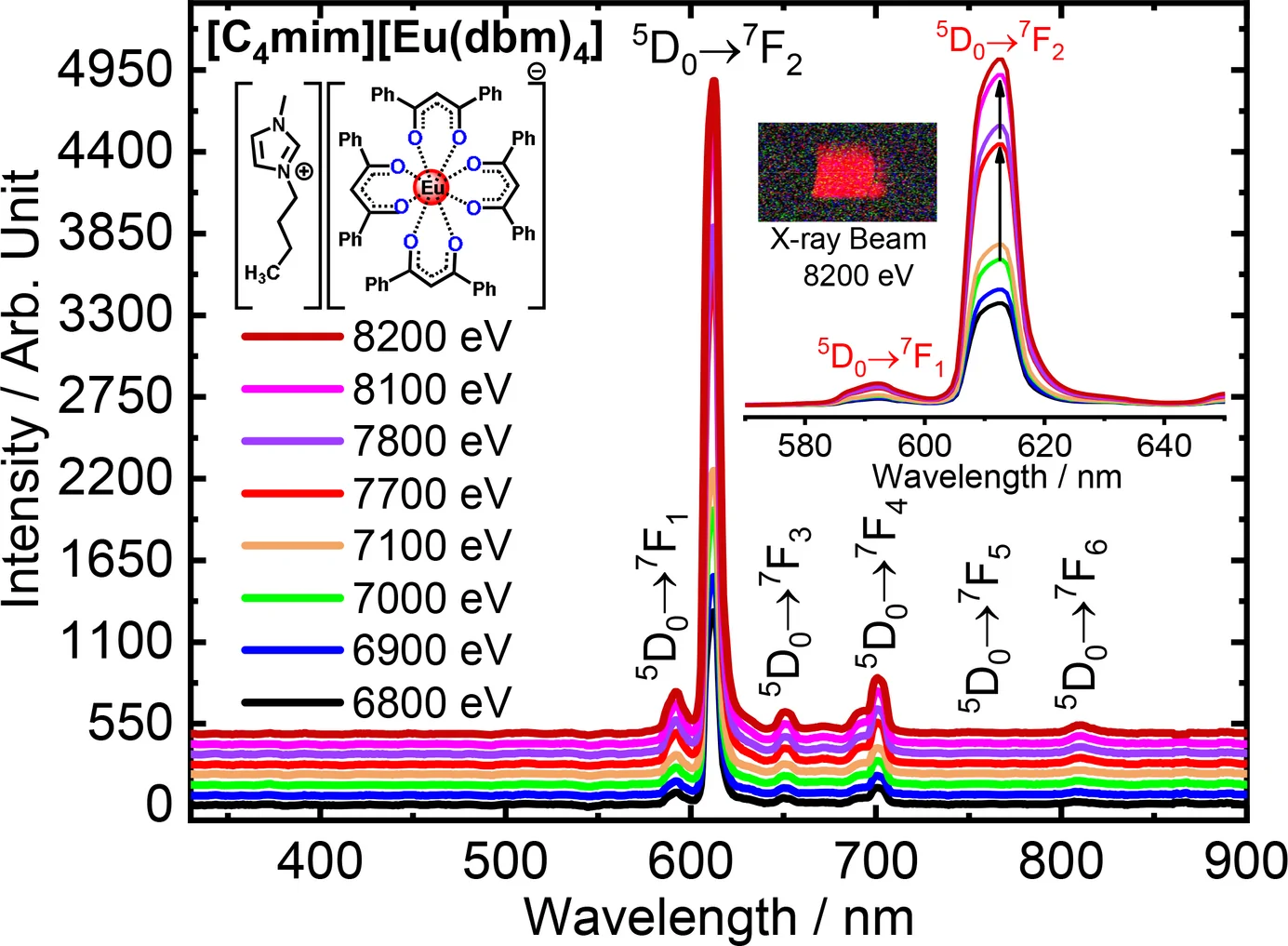
XEOL spectra of the [C_4_mim][Eu(dbm)_4_] complex, measured under excitation at various monochromatic X-ray energies, around the Eu *L*_3_ (6977 eV), *L*_2_ (7617 eV) and *L*_1_ (8052 eV) edges. The inset figures show the chemical structure, magnification of the spectrum in the region of the ^5^D_0_ → ^7^F_1,2_ emission lines and a photoimage of the red emitting scintillator captured by the built-in Basler GigE camera, under an X-ray beam (X-ray beam energy: 8200 eV).

The Eu^3+^ complex was synthesized and reported earlier by our research group at the Institute of Chemistry, University of Sao Paulo (USP), Brazil (Khan *et al.*, 2023 ▸). These highly efficient X-ray-induced luminescent rare-earth complexes could be remarkable alternatives for crystalline thin-film scintillators, such as GGG:Eu. These complexes can be easily mixed with polymer to prepare a desired homogenous emission color and thickness for thin-film scintillators employed in high-energy detection systems, such as X-ray computed tomography (XCT) experimental setups. To explore the X-ray scintillation properties of the [C_4_mim][Eu(dbm)_4_] complex, where dbm refers to di­benzoyl­methane and C_4_mim refers to 1-butyl-3-methyl­imidazolium bromide, an XEOL study was performed and a photoimage of the scintillator powder was captured via a built-in Basler GigE camera, under irradiation with an X-ray beam of 8200 eV energy (inset, Fig. 4 ▸). This Eu^3+^ complex demonstrated remarkably higher intensity red emission when compared with the traditional single-crystal GGG:Eu scintillator (Fig. 2 ▸), as evident from the high-intensity XEOL emission spectra and the red fluorescence digital image (Fig. 4 ▸) of the [C_4_mim][Eu(dbm)_4_] complex.

XEOL spectra of the [C_4_mim][Eu(dbm)_4_] complex (Fig. 4 ▸) were measured at room temperature (∼300 K) under excitation with a monochromatic X-ray beam of selective energies (6800–8200 eV) around the Eu *L*_3,2,1_ edges. Each spectrum was collected by averaging the ten accumulated scans using the XEOL spectrometer with an integration time of 5 s. These spectra exhibited narrow emission lines assigned to the ^5^D_0_ → ^7^F_*J*_ transitions (where *J* = 1–6) of the Eu^3+^ ion, dominated by the ^5^D_0_ → ^7^F_2_ hypersensitive transition (Teixeira *et al.*, 2019 ▸). The narrow bands and relative intensities of the Eu^3+^ emission lines (^5^D_0_ → ^7^F_1–4_ transitions) allow one to effectively probe and gain insight into the local chemical environment of the Eu^3+^ site (Binnemans, 2015 ▸). The considerable high intensity of the hypersensitive ^5^D_0_ → ^7^F_2_ FED transition (Fig. 4 ▸) suggested a non-centrosymmetric Eu^3+^ site of lower symmetry for the [C_4_mim][Eu(dbm)_4_] compound (Binnemans, 2015 ▸), as the local structure of the compound was explored in detail earlier by our research group, employing quantitative XANES and EXAFS analyses (Khan *et al.*, 2023 ▸).

The emission intensity of the ^5^D_0_ → ^7^F_2_ FED transition increases significantly upon excitation of the Eu 2*p* core electrons, achieved by irradiating with X-ray beam energies of 7700 eV (above the *L*_2_ edge) and 8100 eV (above the *L*_1_ edge), as indicated by the black arrows in the inset of Fig. 4 ▸. This observation suggests an efficient contribution of the 2*p*_1/2_ and 2*p*_3/2_ core-level excitations to the sensitization pathways of the Eu^3+^ intraconfigurational 4*f* emissions. Furthermore, the Eu^3+^ complex exhibits appreciable emission even under excitation with X-ray beam energies below the Eu 2*s* and 2*p* absorption edges, indicating that lower-energy electronic states of Eu also contribute to the sensitization mechanisms responsible for the characteristic red emission [see Fig. S2(*b*)]. Nevertheless, the progressive enhancement in emission intensity with increasing X-ray beam energy (Fig. 4 ▸) demonstrates that [C_4_mim][Eu(dbm)_4_] is a promising visible red-emitting organic scintillator, capable of efficient luminescence over a broad hard X-ray energy range.

#### 3.2.1. Eu^3+^ complex/polymer hybrid films

To explore the optical detection behavior of the XEOL probe at BM08 toward fluorescence thin films, RE^3+^ complex added polymethyl­methacrylate (PMMA) thin films were studied. The tetra­kis-Eu^3+^ complex with the β-diketonate ligand 4,4,4-tri­fluoro-1-(2-naphthyl)-1,3-butane­dione (nta) was synthesized following a previously reported method (Bruno *et al.*, 2009 ▸). In a typical synthesis, EuCl_3_·6H_2_O was dissolved in a methanol/water mixture, and a methano­lic solution of deprotonated nta using NaOH was added dropwise (5:1 of nta^−^:Eu^3+^). Subsequently, a methano­lic solution of tetra­ethyl­ammonium bromide (Et_4_NBr) was added. The reaction mixture was stirred at 60°C for 3 h. The resulting precipitate was filtered, dried in a vacuum desiccator, and recrystallized by layering hexanes over a di­chloro­methane solution to yield transparent crystals. The formation of the Et_4_N[Eu(nta)_4_] complex (see Fig. S3) was confirmed by elemental analysis (CHN): calculated and found values were C 57.24% (57.10%), H 3.90% (3.83%) and N 1.04% (1.13%), respectively. Furthermore, confirmation of the mass of the Et_4_N[Eu(nta)_4_] complex was obtained using electrospray ionization mass spectrometry (MicroTOF ESI^+^). The mass-spectroscopy analysis (see Fig. S4) revealed a peak at *m*/*z* ≃ 1213.1200, which is assigned to the [C_56_H_32_EuF_12_O_8_-M]^−^­[Et_4_N]^+^ species, agreeing closely with its calculated value (*m*/*z* = 1213.1123).

**Figure 5:**
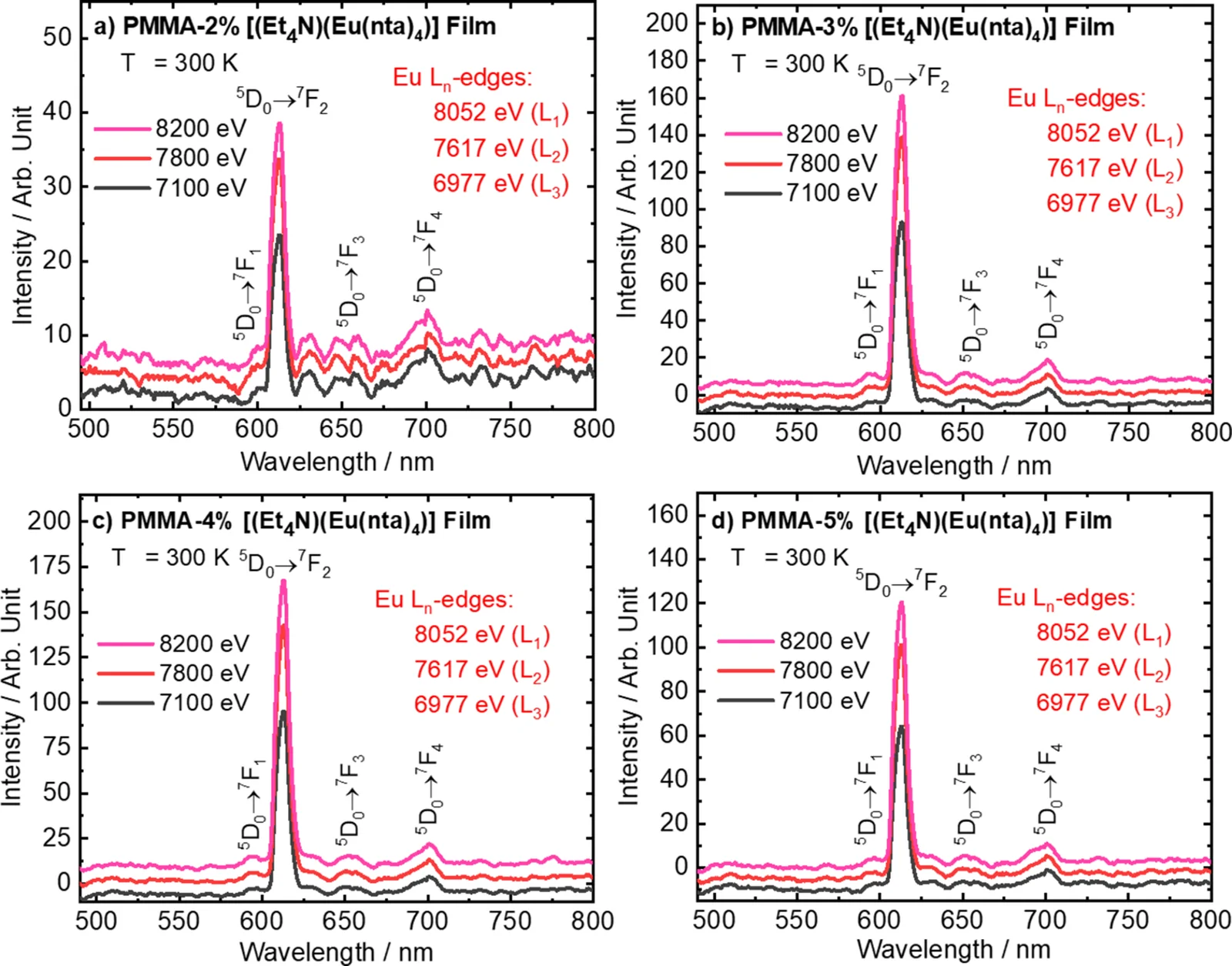
(*a*)–(*d*) XEOL spectra of the PMMA–(2–5%)Et_4_N[Eu(nta)_4_] hybrid films, measured under excitation at 7100, 7800 and 8200 eV monochromatic X-ray energies, above the Eu *L*_3,2,1_ edges.

The hybrid films of PMMA polymer doped with varying loading concentrations of 2–5 wt% of Et_4_N[Eu(nta)_4_] complex were prepared according to the literature-reported procedure (Kai *et al.*, 2011 ▸). Briefly, the PMMA was dissolved in acetone, followed by the addition of the Et_4_N[Eu(nta)_4_] complex at different weight percentages (2%–5%) to give a transparent homogeneous solution. Finally, the solutions were carefully drop-casted onto 2 × 5 cm glass substrates in thin layer forms. The PMMA–Et_4_N[Eu(nta)_4_] hybrid films (see Fig. S5) were then dried under a saturated atmosphere to prevent opacity. XEOL spectra of the PMMA–Et_4_N[Eu(nta)_4_] hybrid films (Fig. 5 ▸) were measured at room temperature (∼300 K) under excitation with a monochromatic X-ray beam of selective energies 7100, 7800 and 8200 eV (above the Eu *L*_3,2,1_ edges). Each spectrum was collected by averaging ten accumulated scans using the XEOL spectrometer with an integration time of 5 s. These spectra exhibited narrow emission lines assigned to the ^5^D_0_ → ^7^F_*J*_ transitions (where *J* = 1–4) of the Eu^3+^ ion, dominated by the ^5^D_0_ → ^7^F_2_ hypersensitive transition (Teixeira *et al.*, 2019 ▸).

The high emission intensity of the hypersensitive ^5^D_0_ → ^7^F_2_ FED transition (Fig. 5 ▸) suggested a non-centrosymmetric Eu^3+^ chemical environment for all the PMMA–(2,3,4,5%)Et_4_N­[Eu(nta)_4_] films. A continuous increase in Eu emission intensity was observed by increasing the concentration of the Et_4_N[Eu(nta)_4_] photoemitting complex in hybrid films from 2 to 4 wt% (Fig. 5 ▸). However, a decrease in Eu emission intensity for the PMMA–(5%)Et_4_N[Eu(nta)_4_] film was observed, suggesting concentration quenching, which might be due to the cross-relaxation process among the Eu^3+^ ions. Nevertheless, the XEOL study suggests that hybrid thin films could be next-generation organic scintillators for ionizing radiation detection and imaging applications, such as XCT.

### 3.3. XEOL study of quantum dots

**Figure 6:**
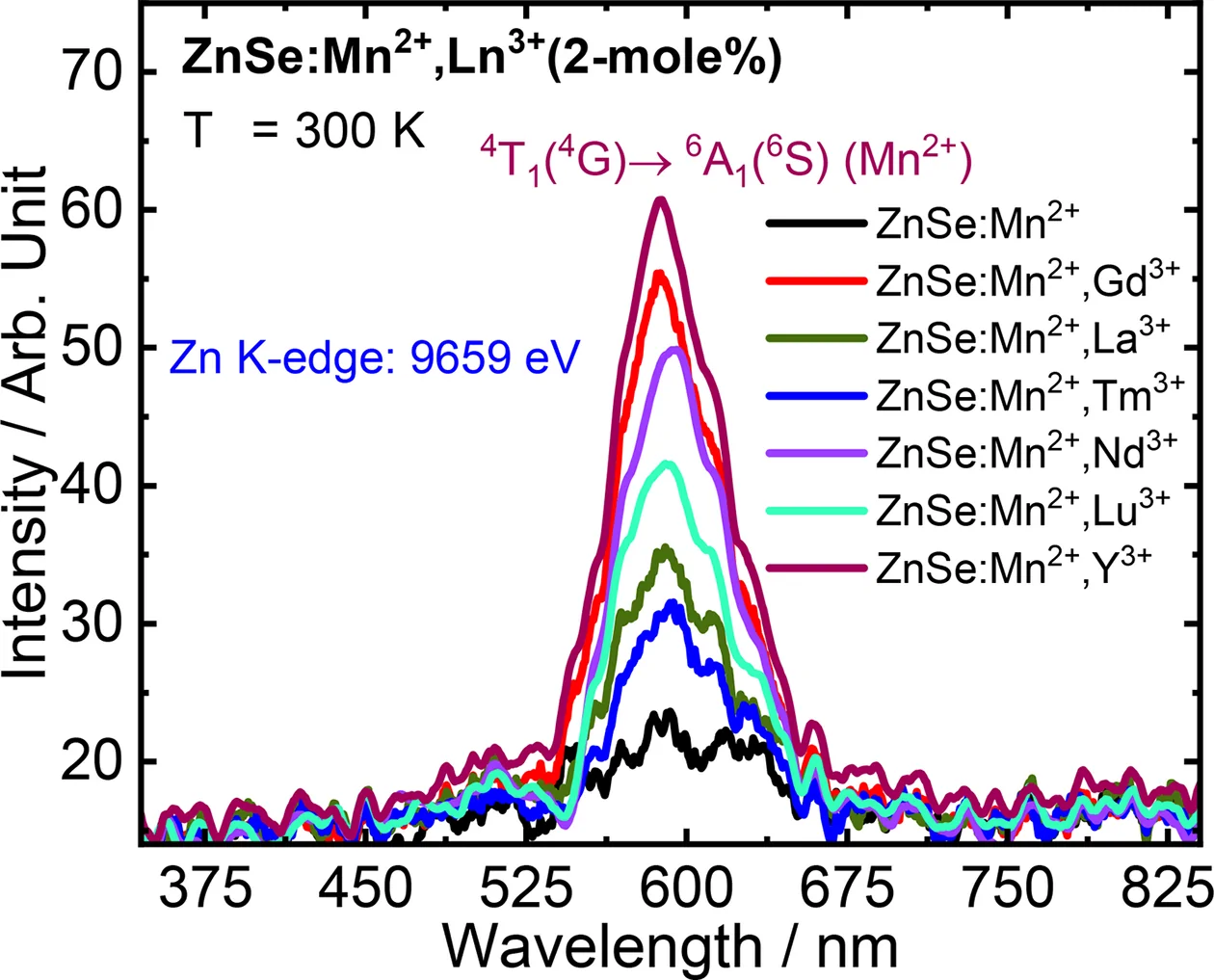
XEOL spectra of the RE^3+^-doped ZnSe:Mn^2+^ QDs measured under excitation at monochromatic X-ray beam energy of 9700 eV, above the Zn *K* edge.

The capability of the XEOL setup at the BM08-XAFS/XRF beamline to probe the optoelectronic properties of semiconductor QDs was systematically investigated. Trivalent rare-earth ion (RE^3+^) doped ZnSe:Mn^2+^ QDs were previously synthesized and reported (Khan *et al.*, 2022 ▸; Gul, Khan, Khan *et al.*, 2025 ▸) by our research group at the Institute of Chemistry, USP, Sao Paulo, Brazil. XEOL spectra (Fig. 6 ▸) of these representative QD samples were measured at room temperature over the 200–950 nm spectral range using the XEOL spectrometer at BM08. Each spectrum represents the average of five accumulated scans with an integration time of 5 s per scan. XEOL spectra of the QDs exhibited a broad peak at ∼590 nm assigned to the ^4^T_1_(^4^G) → ^6^A_1_(^6^S) transition (Yang *et al.*, 2019 ▸; Gul, Khan, Khan *et al.*, 2025 ▸) of the Mn^2+^ activator ion (Fig. 6 ▸) under excitation with a monochromatic X-ray energy of 9700 eV. A pronounced enhancement in Mn^2+^ emission intensity was observed upon co-doping ZnSe:Mn^2+^ with RE^3+^ ions (particularly Gd^3+^, Y^3+^ and Nd^3+^), which is consistent with the previously reported results (Khan *et al.*, 2022 ▸; Gul, Khan, Khan *et al.*, 2025 ▸; Gul, Khan, Galani *et al.*, 2025 ▸). The energy levels of the RE^3+^ ions are usually located near the host band edges, facilitating the localization of the electron–hole pairs (charge traps) near the dopant sites and enhancing excitation efficiency of the photoemitting Mn^2+^ ions through multiple energy-transfer pathways (Debnath *et al.*, 2020 ▸). In particular, energy transfer from the 4*f* intraconfigurational energy levels of the RE^3+^ ions to the ^4^T_1_-excited Mn^2+^ ions contributes to the observed luminescence enhancement.

Moreover, the 3*d*^5^-intraconfigurational transitions of the Mn^2+^ ion are highly sensitive to the local chemical environment, owing to the strong ligand field effect (Khan *et al.*, 2022 ▸). Therefore, incorporation of RE^3+^ ions into the ZnSe:Mn^2+^ host, which possess relatively larger ionic radii when compared with the Zn^2+^ ion, induces lattice distortion and defects formation (*e.g.* zinc vacancies), arising from the oxidation-state mismatch (charge imbalance) at mixed occupancy RE^3+^/Zn^2+^ sites (Marin & Jaque, 2021 ▸), which can trap electrons. These defects act as electron traps within the band gap; thus, the charge transfer from the electron traps/defects to the Mn^2+^ excited state and suppression of band-edges radiative decay during electron–hole recombination also cause an improvement in the efficiency of photoemission from the activator Mn^2+^ ion.

### 3.4. Detection of Eu impurity and 5*d* → 4*f* emission by XEOL

The XEOL experimental setup at BM08 is a highly sensitive probe for detecting the photoemitting rare-earth ion impurity at an ultrasmall level in the host materials. For instance, Gd_2_O_3_ is an important commercially available heavy rare-earth material, presenting high thermal stability and unique magnetic properties that make it a valuable host for rare-earth ions dopant, such as Nd and Er, used in highly efficient solid-state lasers, telecommunications and medical procedures. In order to gain insight on the purity of the commercially available Gd_2_O_3_ (99.9%) powder, XEOL analysis was employed and detected the Eu impurity in the product. The XEOL spectra (see Fig. S6) of the Gd_2_O_3_ (99.9%) powder (Sigma–Aldrich) were measured at room temperature under excitation with monochromatic X-ray energies above the Eu *L*_1,2,3_ edges (8200, 7800 and 7100 eV), measuring five accumulated scans with an integration time of 5 s for each spectrum. These spectra exhibited narrow emission lines assigned to the ^5^D_0_ → ^7^F_*J*_ transitions (where *J* = 1–4) of the Eu^3+^ ion, dominated by the ^5^D_0_ → ^7^F_2_ hypersensitive transition. In addition, a narrow emission line from the ^6^P_7/2_ → ^8^S_7/2_ transition was also observed in the emission spectrum of Gd_2_O_3_. This result showed the capability and high sensitivity of the XEOL spectrometer to detect efficiently the Eu impurity even in highly pure commercial Gd_2_O_3_. Eu-doped BaAl_2_O_4_ is a widely explored visible-light-emitting phosphor that was previously synthesized and reported (Khan *et al.*, 2025 ▸) by our research group at the Jordan Atomic Energy Commission (JAEC). To demonstrate the efficient spectral resolution of the XEOL spectrometer at the BM08 XAFS/XRF beamline, we presented the result of exploring Eu impurities in a BaAl_2_O_4_ host using the above representative sample.

The XEOL spectra of the Eu-doped BaAl_2_O_4_ (see Fig. S7) were recorded at room temperature under excitation with monochromatic X-ray energies of 7150, 7710 and 8150 eV, corresponding to energies above the Eu *L*_3,2,1_ edges. These spectra exhibit broad emission bands at a maximum of ∼508 nm with a shoulder peak at ∼495 nm (see Fig. S7), attributed to the 4*f*^6^5*d*^1^ → 4*f*^7^ interconfigurational transitions of the Eu^2+^ ion (Teixeira *et al.*, 2019 ▸). Furthermore, sharp emission lines were clearly resolved and assigned to the ^5^D_0_ → ^7^F_*J*_ (where *J* = 1, 2, 3, 4 and 6) transitions of the Eu^3+^ ion, dominated by the ^5^D_0_ → ^7^F_2_ hypersensitive transition. The clear differentiation between the broad Eu^2+^ emission band and narrow Eu^3+^ emission lines highlighted the remarkable spectral resolution of the XEOL setup. This finding further suggested incorporation of Eu^2+^/Eu^3+^ ions at two nonequivalent Ba sites in the BaAl_2_O_4_:Eu phosphor, consistent with our previous detailed report (Khan *et al.*, 2025 ▸).

**Figure 7:**
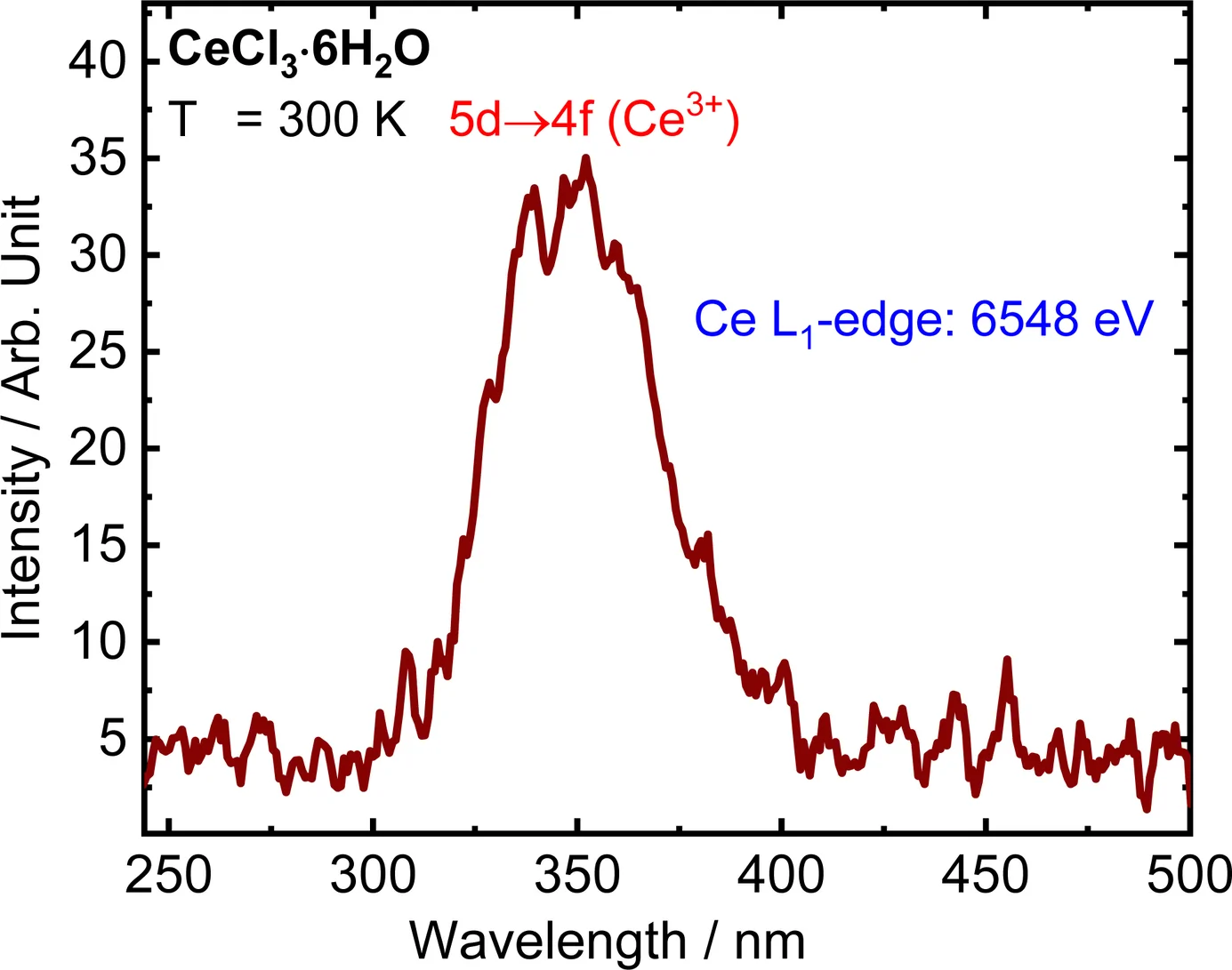
XEOL spectrum of CeCl_3_·6H_2_O, measured under excitation at 6700 eV X-ray photon energy, above the Ce *L*_1_ edge.

Ce is a rare-earth element with remarkable applications in display and lighting technologies, for instance Y_3_Al_5_O_12_:Ce^3+^ (YAG:Ce^3+^) in white-light-emitting diodes (wLEDs). The UV emission from the 5*d* → 4*f* interconfigurational transition of the Ce^3+^ ion was efficiently detected using the XEOL spectrometer at the BM08-XAFS/XRF beamline. The XEOL spectrum (Fig. 7 ▸) of CeCl_3_·7H_2_O (Sigma–Aldrich) was measured at room temperature under excitation with monochromatic X-ray energy above the Ce *L*_1_ edge (6548 eV), measuring five accumulated scans with an integration time of 5 s per scan. This spectrum (Fig. 7 ▸) displayed broad bands corresponding to the interconfigurational transition from the lowest Ce^3+^ 5*d* level to the two spin–orbit components of the ^2^F_5/2,7/2_ ground state. This result demonstrates the capability of the XEOL spectrometer to efficiently measure the optical photon emission from materials in the UV region.

### 3.5. XEOL spectral mapping

XEOL and laser-excited fluorescence 2D mapping provide a multimodal experimental probe for the spatially resolved investigation of optical emission from materials under element-specific monochromatic X-ray and UV–Vis/NIR laser excitations. The step-by-step or raster-scanned 2D spectral mapping allows visualization of spatial heterogeneities in optical emission, defect distributions and photoemitting site gradients in optical materials/thin films at the micrometre-scale level, which is often inaccessible with conventional bulk optical spectroscopy techniques. XEOL and laser-excitation 2D spectral mappings are significant for optical characterizations of advanced functional materials such as QDs, rare-earth phosphors and lanthanide complexes, where local structure and chemical environment of the photoemitting sites critically influence the optical performance and emission efficiency of materials, which are crucial in photonic device functionality.

**Figure 8:**
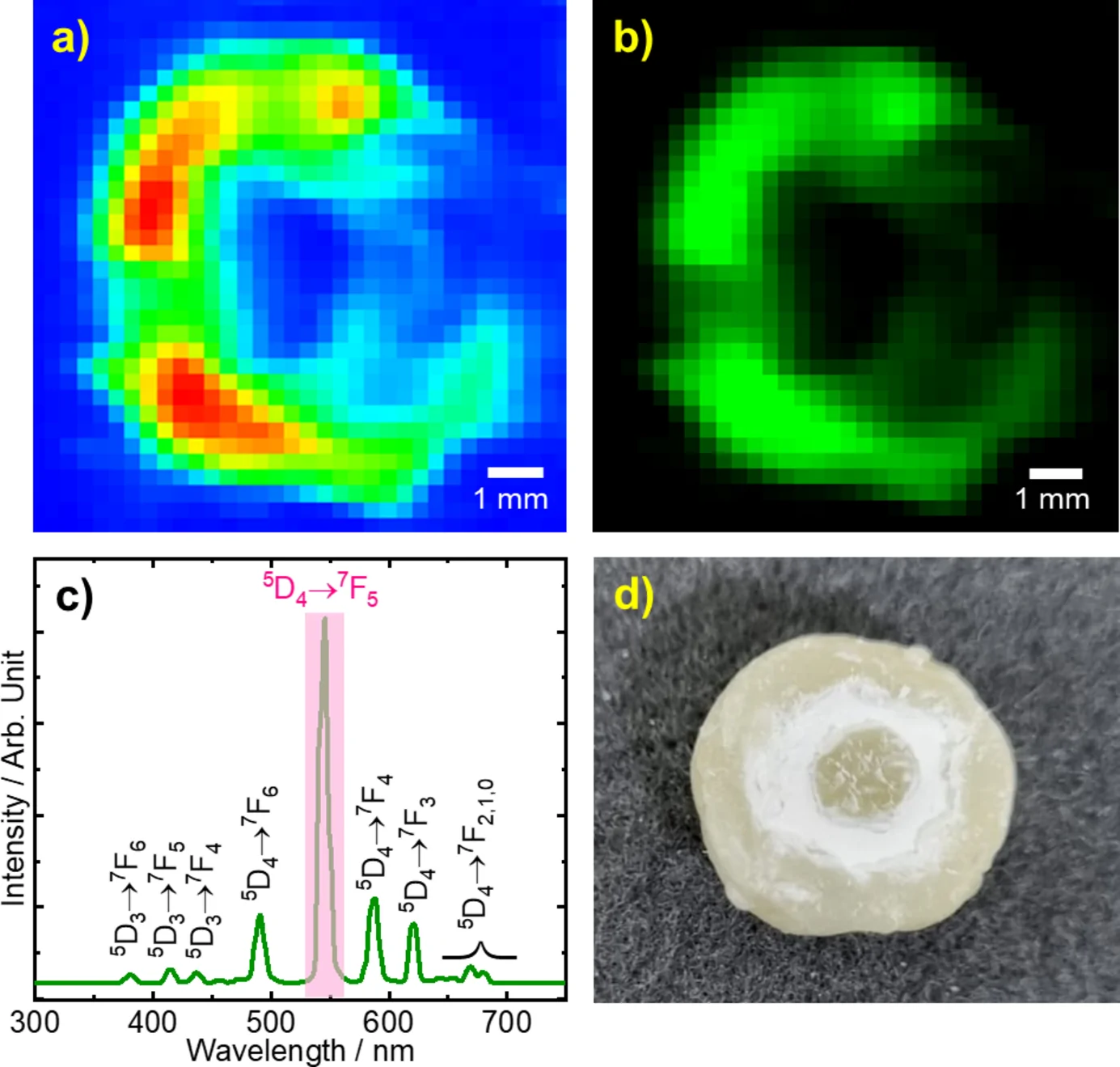
XEOL spectral mapping of Gd_2_O_2_S:Tb phosphor recorded under monochromatic X-ray excitation at 9000 eV. (*a*) Original spectral stack collected across the scanned area and (*b*) 2D spectral map constructed by integrating the selected ROI in the emission spectrum corresponding to the Tb^3+^^5^D_4_ → ^7^F_5_ transition (∼545 nm), revealing the spatial distribution of the green-emitting Tb^3+^ centers in the sample. (*c*) XEOL spectrum with the defined ROI of the ^5^D_4_ → ^7^F_5_ transition that is used for constructing 2D mapping. (*d*) Digital photograph of the Gd_2_O_2_S:Tb-containing sample.

To evaluate the performance of the newly developed XEOL mapping tool, including motion accuracy of the BM08’s sample stage and the synchronization of the XEOL spectrometer, a step-by-step XEOL mapping was performed on a representative solid wax sample embedded with a concentric ring structure of Gd_2_O_2_S:Tb phosphor on its surface (Fig. 8 ▸). XEOL 2D spectral mapping was performed over a 10 × 10 mm area of the representative sample to spatially resolve the characteristic emission of Tb^3+^ ions in a phosphor under X-ray excitation. The sample was rotated at 45° to the incident beam, and the optical fiber coupled to a collimating lens at its end was positioned at 90° with respect to the X-ray beam axis. XEOL mapping data were acquired at 9000 eV (X-ray beam energy) in step-by-step scanning mode with a step size of 200 µm in both horizontal and vertical directions and an integration time of 1.0 s. The X-ray beam was slit down to the extent that the beam intensity effectively disappeared upon an additional 200 µm slit closure, ensuring a tightly confined beam footprint and micrometre spatial resolution.

The full XEOL emission spectrum was recorded at each pixel position, enabling extraction of the Tb^3+^ emission and quantitative analysis of its spatial distribution in the mapping area. The complete spectral dataset was saved in HDF5 format as a spectral stack, and the 2D luminescence distribution maps were reconstructed and visualized using the region of interest (ROI) imaging tool in *PyMca*, which is open software. The distribution of the photoemitting Tb^3+^ site was mapped on the basis of its dominant high-intensity ^5^D_4_ → ^7^F_5_ transition (∼545 nm) via defining an ROI of the corresponding peak in the emission spectrum (Fig. 8 ▸). The reconstructed spectral map clearly reveals the spatial distribution of Tb^3+^ as a green emission map across the scanned area in the form of concentric ring patterns, correlating with the digital image captured through the XEOL camera, which validates the success of the XEOL mapping tool and the localization of the photoemitting Tb^3+^ ions in the sample (Fig. 8 ▸).

The image map is derived from spectrally resolved emission lines of the Tb^3+^ ions rather than broad-band intensity, this approach provides chemically and optoelectronically specific spatial information that correlates the luminescence with the local electronic structure of the photoemitting sites in the phosphor.

#### 3.5.1. Laser-excited hyperspectral fluorescence mapping

**Figure 9:**
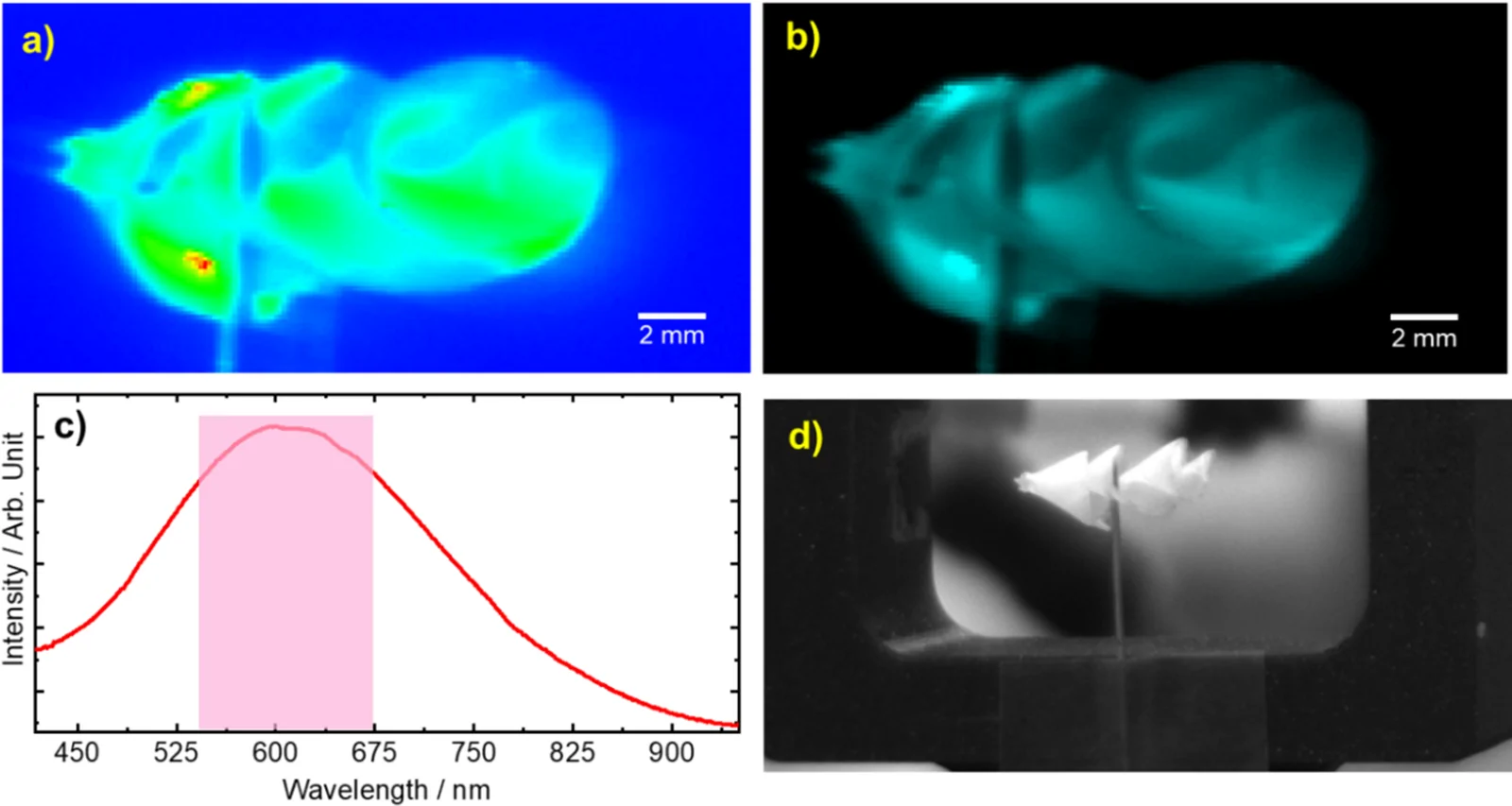
Hyperspectral mapping of orange fluorescence spiral-shaped polyethyl­ene block measured under 400 nm laser excitation. (*a*) Original spectral stack collected across the scanned area and (*b*) 2D spectral map constructed by integrating the selected ROI in the fluorescence band, using the ROI imaging tool in *PyMca*. (*c*) Fluorescence spectrum with the defined ROI used for constructing the 2D spectral mapping. (*d*) Digital photograph of the spiral-shaped polyethyl­ene block.

To evaluate the achievable spatial resolution, two-dimensional laser-excited fluorescence mapping was conducted on a fluorescence polyethyl­ene block patterned in a spiral shape (Fig. 9 ▸), commonly used for radiation safety purposes. The hyperspectral-mapping measurements were performed in step-by-step scanning mode using a 400 nm excitation laser with a spot size of ∼200 µm. This specific geometry was adopted for the spectral mapping to minimize the laser coherent scattering. The sample was oriented at 45° to the incident laser beam, and the optical fiber coupled to a collimating lens and 420 nm longpass filter at its end was positioned at 90° with respect to the laser-beam axis to reduce the collection of elastically scattered light. The mapping scan was acquired with a step size of 100 µm and an integration time of 50 ms per point, using the XEOL spectrometer. The 420 nm longpass filter was employed to suppress coherent laser scattering originating from the sample. The acquired hyperspectral fluorescence dataset was saved in HDF5 format and analyzed using the ROI imaging tool in *PyMca*.

The hyperspectral data were visualized as a spectral stack, from which an ROI at a maximum of 607 nm, corresponding to the fluorescence emission band (Fig. 9 ▸) of the sample, was selected. This spectral ROI was integrated to generate a two-dimensional intensity distribution map using the ROI imaging tool of *PyMca*. The reconstructed image clearly resolves the spiral geometry of the polyethyl­ene sample without noticeable artefacts, demonstrating accurate synchronization between the sample-stage motion and the spectrometer acquisition system. These results confirm that the XEOL probe is fully capable of performing fluorescence spectral mapping under selective laser excitation in the UV–Vis to NIR spectral region, covering both downconversion and upconversion luminescence processes.

## 4. Conclusions

An advanced XEOL experimental setup was successfully developed and commissioned at the BM08-XAFS/XRF beamline of SESAME. The system provides a highly sensitive spectroscopic probe for the user community, enabling combined XEOL and XAS measurements for the investigation of a broad range of optical materials. X-rays are a clean energy that avoid the stray scattering of UV–Vis light, contrary to the PL study, and penetrate the material deeper. These features make XEOL a highly selective and bulk-sensitive technique to precisely probe the optical emission from materials and crucially understand the electronic structure, defect states/charge traps and energy-transfer processes within the material. Multiple optical materials were studied using the developed probe, including luminescence spectral measurements and two-dimensional spectral mapping, demonstrating high sensitivity, reliable synchronization and micrometre spatial resolution under laser irradiation. This work highlighted the strength of XEOL as a powerful technique for elucidating the optoelectronic structure and luminescence mechanisms in optical materials under irradiation with X-ray and laser sources. The capability to achieve site selectivity through the combination of XAS and XEOL is particularly significant for optimizing optical materials for advanced applications, including display and lighting technologies, and scintillators used in X-ray detection systems for medical imaging modalities such as computed tomography.

## 5. Related literature

The following reference is cited in the supporting information for this article: Phatrapornnant & Pont (2006 ▸).

## Supplementary Material

Supporting information. DOI: 10.1107/S2052252526007815/ro5048sup1.pdf
