# Supplementary material for: Lighting up materials with X-rays: advanced X-ray excited optical luminescence probe at the BM08-XAFS/XRF beamline, SESAME
Source: IUCrJ. 2026 Oct 6;13(Pt 6):705–16. doi: 10.1107/S2052252526007815 (PMC13763899; doi:10.1107/S2052252526007815)
Supplement: Supplementary file 1 [file m-13-00705-sup1.pdf]

IUCrJ

Volume 13 (2026)

Supporting information for article:

Lighting up materials with X-rays: advanced X-ray excited optical luminescence probe at the BM08-XAFS/XRF beamline, SESAME

Latif U. Khan, Abdel R. Hasoneh, Abdalla Ahmad, Can Dokuyucu, Anas Mohammad, Allayth Aldrabee, Muhammad Iqbal, Farouq Al Omari, Almuatasem Al Natsheh, Abid U. Rehman, Maher Shehab, Mustafa AlZubi, Hussein Al-Mohammad, Zahid U. Khan, Felipe da S. M. Canisares, Messaoud Harfouche, Santiago J. A. Figueroa, Fareeha Hameed, Andrea Lausi and Hermi F. Brito

S1. Design of Trigger Generator

Trigger generator is an in-house development device that is built with available components to Tr. Design of the device based on PIC microcontroller (16F877A) and communicates via the RS-485 protocol using a MAX485 converter module. Device can generate 8 independent hardware trigger signals controlled via SCPI commands. Trigger outputs support CMOS, TTL, and dry contact configurations. Main purpose of the device in the experiment, is to generate software controlled arbitrary trigger for the spectrometer. This allows the experiment timing to be set with any script. Software based trigger systems usually have respectively high timing jitter on their output (µs to ms) but due to large timing tolerance of the experiment, output jitter is reasonable (Phatrapornnant & Pont, 2006). Output pulse width values can be set between 1.0 ms to 9999 ms for gated or triggered operation. Status LEDs for each channel illuminate when the respective channel is triggered, and the trigger status LED will light up after receiving a trigger command from the control system.

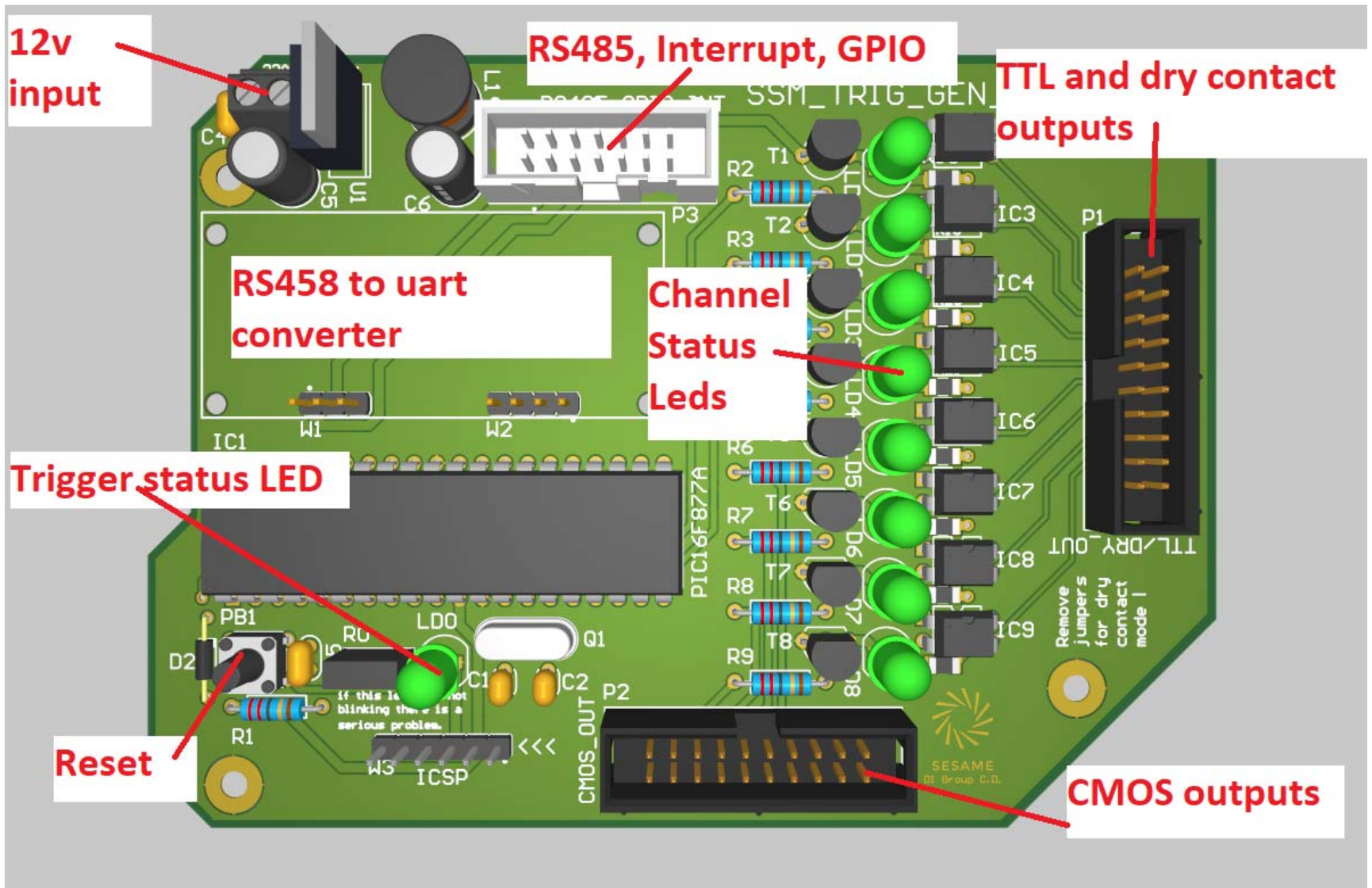

Figure S1 Trigger generator designed in-house for XEOL spectrometer, generating hardware trigger with EPICS control system.

S2. XEOL Mechanism in $Gd_2O_2S:Tb^{3+}$ and $[C_4mim][Eu(dbm)_4]$ X-ray Scintillators

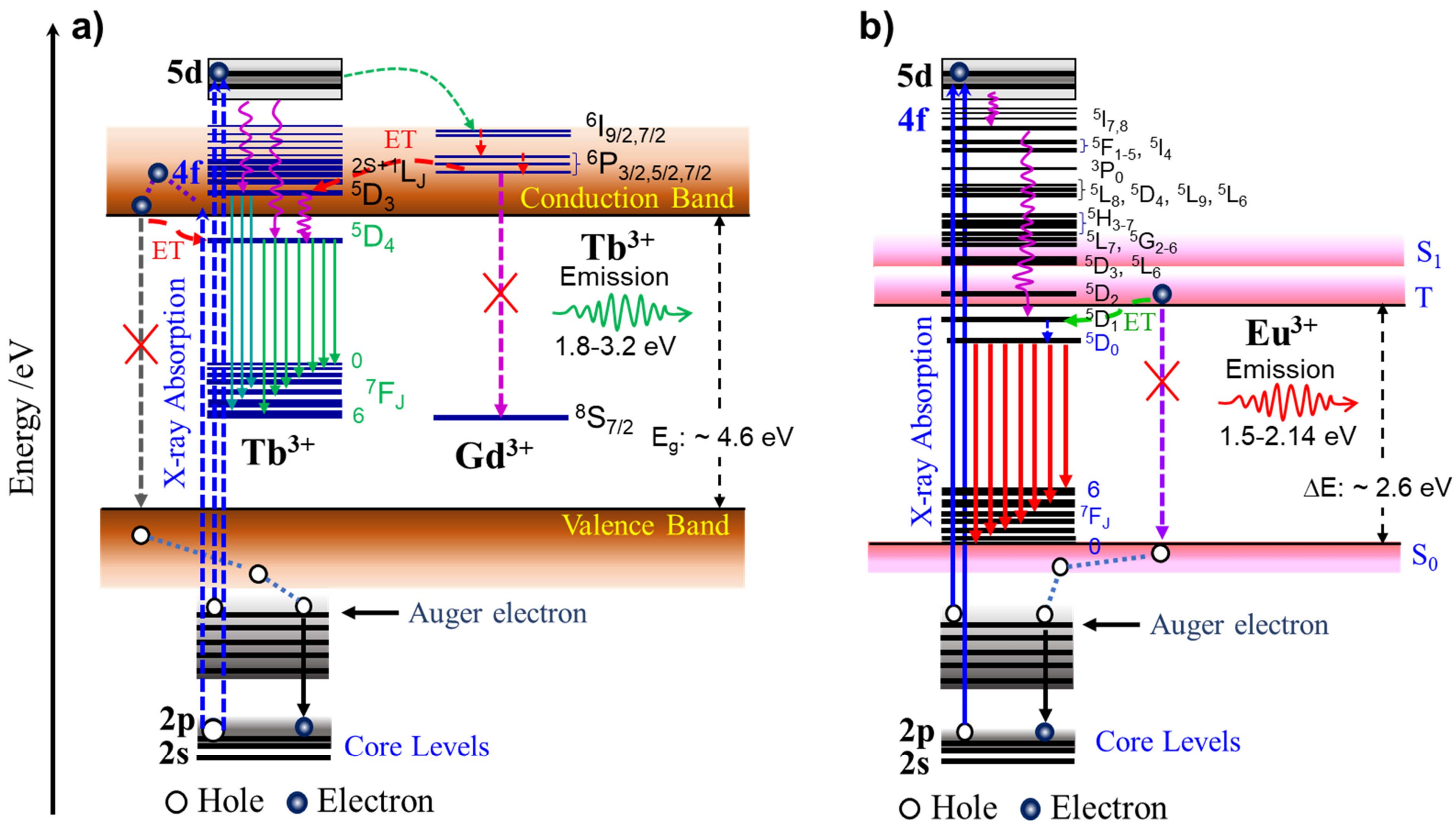

Figure S2 Possible energy transfer (ET) mechanism of X-ray induced luminescence in $Gd_2O_2S:Tb^{3+}$ (a) and $[C_4mim][Eu(dbm)_4]$ (b) phosphors. ET diagram involves monochromatic X-ray absorption by Eu^{3+}, Gd^{3+} and Tb^{3+} ions (p→d transition) and generation of core-hole, an electron and core-hole recombination through complex energy transfer pathways, following the sensitization/excitation of photoemittiong Tb^{3+} and Eu^{3+} 4*f* energy levels, which decays to the fundamental energy levels by emitting optical photons.

S3. Synthesis of $Et_4N[Eu(nta)_4]$ Complex

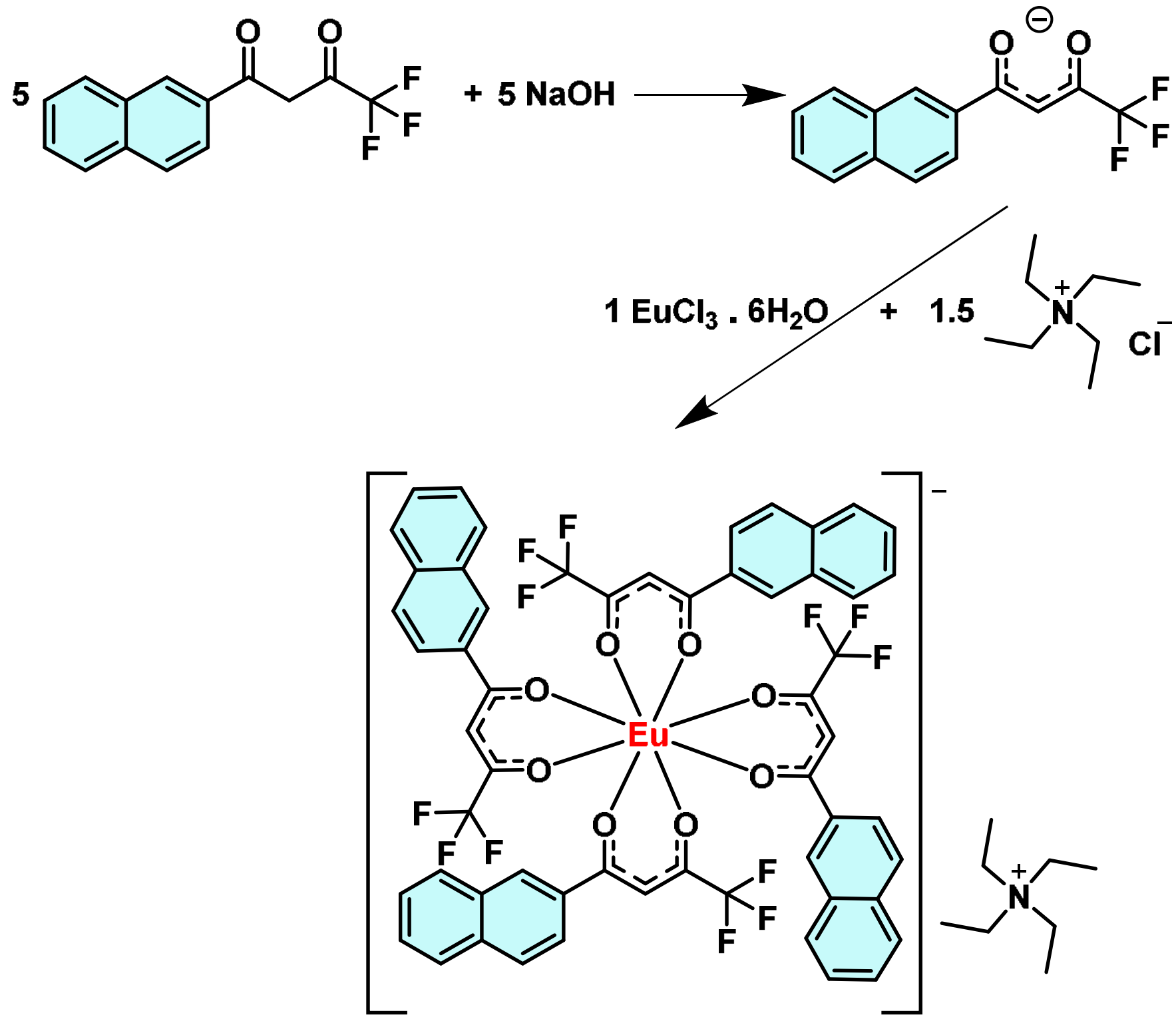

Figure S3 Synthesis scheme of the $Et_4N[Eu(nta)_4]$ complex, Et: tetraethylammonium, Eu: Europium and nta: 4,4,4-trifluoro-1-(2-naphthyl)-1,3-butanedione.

S4. Mass Spectrum of $Et_4N[Eu(nta)_4]$ Complex

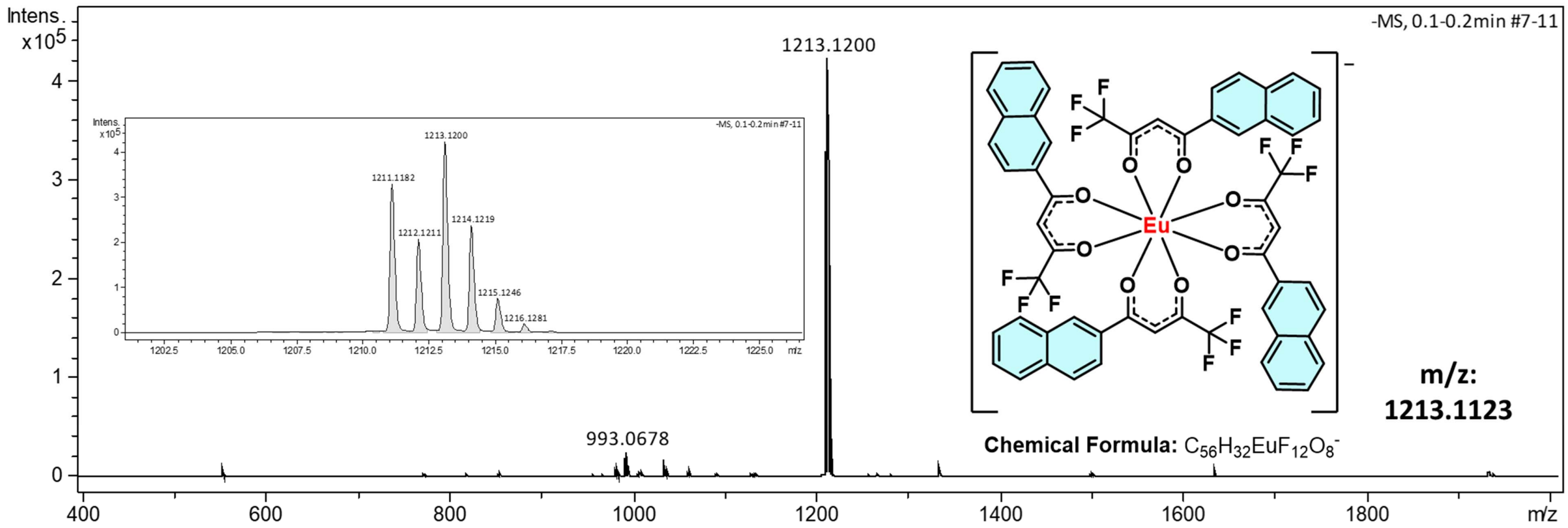

Figure S4 Mass spectrum of $Et_4N[Eu(nta)_4]$ complex. The insets figures display the magnified view of the main peak at m/z: ~1213 and chemical structure of the $Et_4N[Eu(nta)_4]$.

S5. Eu Complex / PMMA hybrid Films

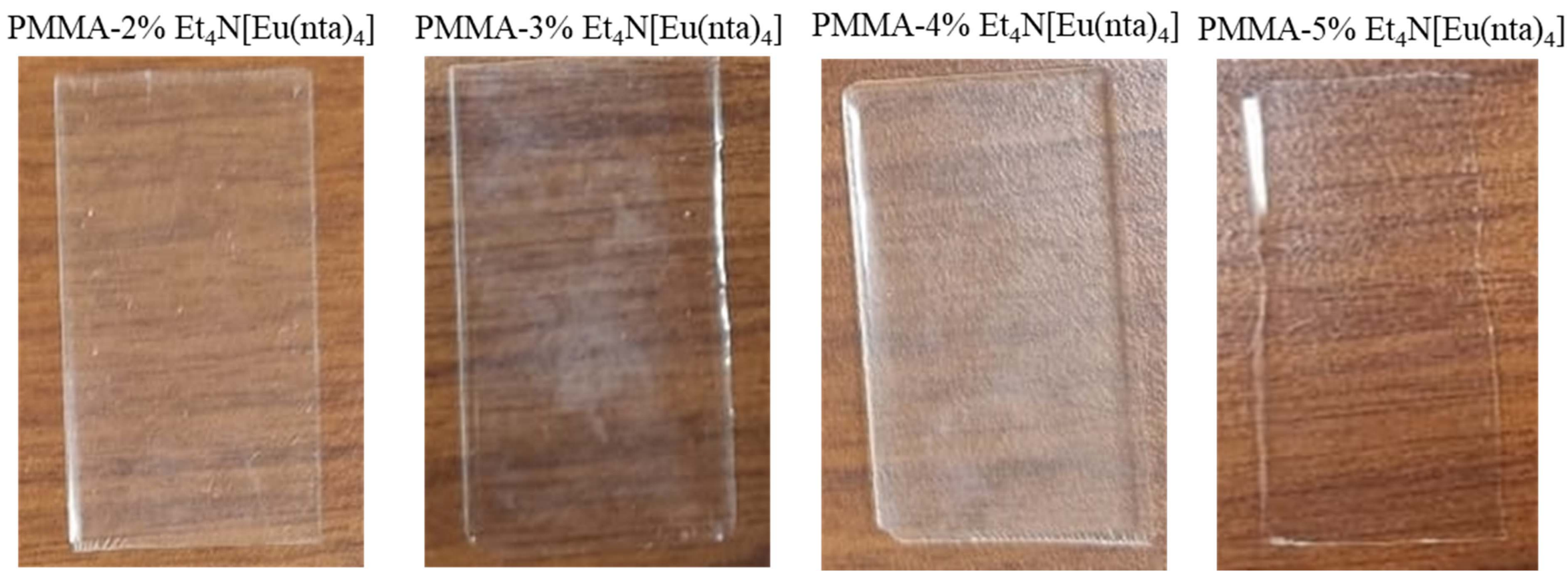

Figure S5 Photographs of hybrid films (from left to right) prepared by doping 2-5% of the $Et_4N[Eu(nta)_4]$ complex in polymethylmethacrylate (PMMA) polymer.

S6. XEOL Spectra of Commercial Gd_2O_3 (99.9%)

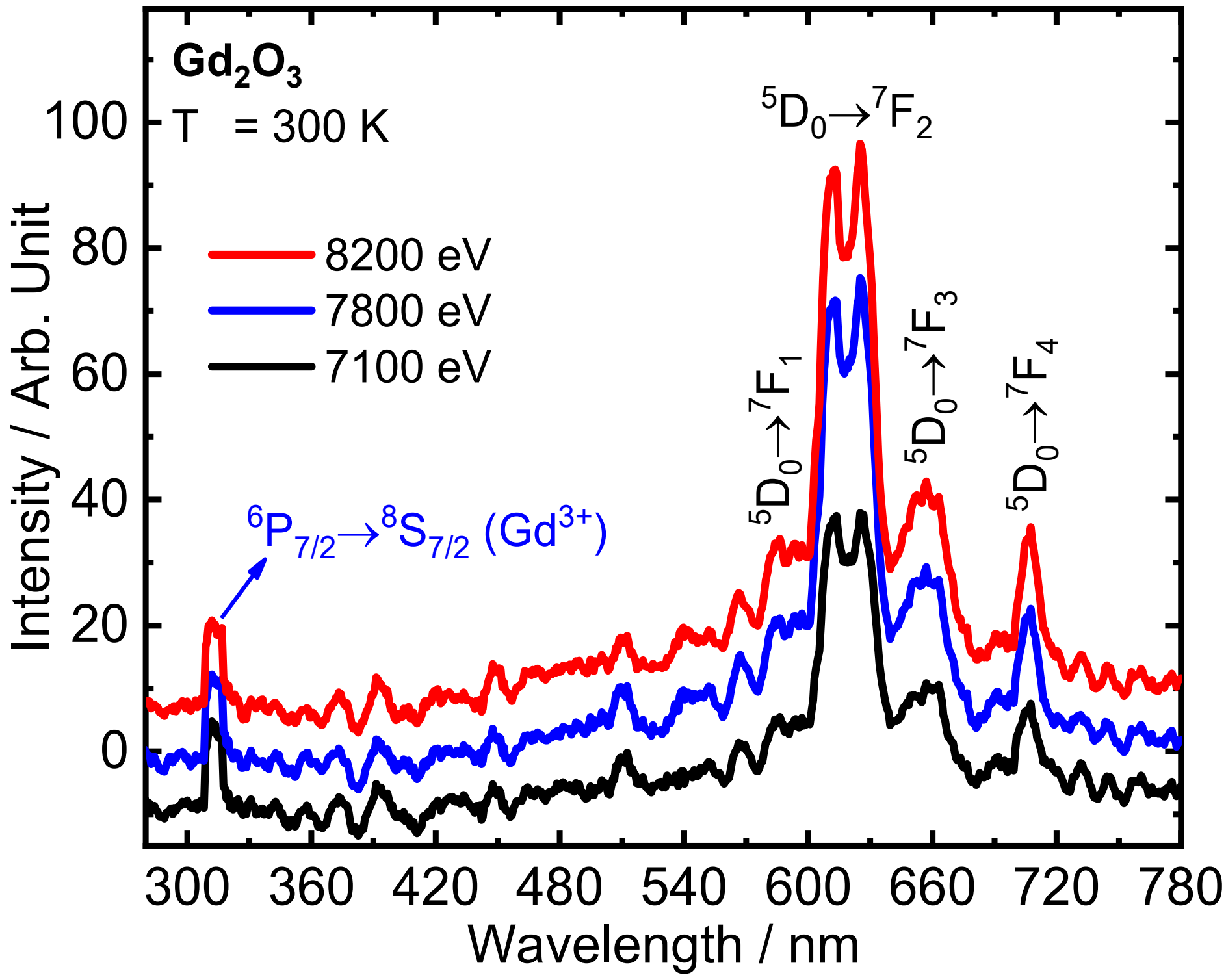

Figure S6 XEOL spectra of the commercial Gd_2O_3 (99.9 %), measured under excitation at 7100, 7800 and 8200 eV X-ray energies, above the Eu $L_{3,2,1}$-edges (6977, 7617 and 8052 eV).

S7. XEOL Spectra of $BaAl_2O_4:Eu^{2+/3+}$ Phosphor

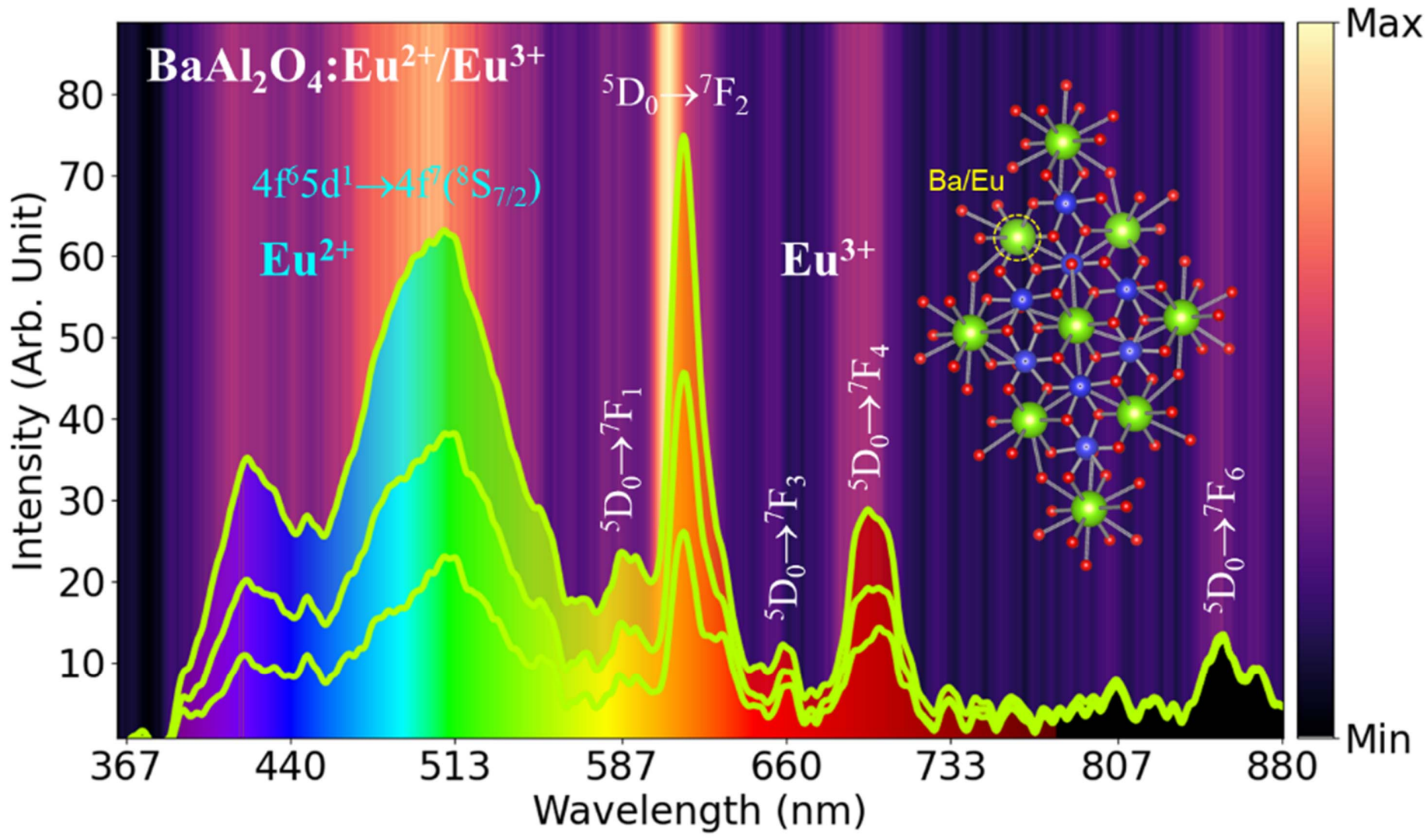

Figure S7 XEOL spectra of the $BaAl_2O_4:Eu^{2+/3+}$ phosphor (3D structure in inset figure), measured under excitation at 7150, 7710 and 8150 eV X-ray photons energy, above the Eu $L_{3,2,1}$-edges.

S8. References

Phatrapornnant, T. & Pont, M. J. (2006). *IEEE Transactions on Computers* **55,** 113-124.
